# Maternal dietary antioxidant supplementation regulates weaned piglets’ adipose tissue transcriptome and morphology

**DOI:** 10.1371/journal.pone.0310399

**Published:** 2024-09-12

**Authors:** Hernán D. Laviano, Gerardo Gómez, Yolanda Núñez, Juan M. García-Casco, Rita M. Benítez, Ana de las Heras-Molina, Fernando Gómez, Fernando Sánchez-Esquiliche, Beatriz Martínez-Fernández, Antonio González-Bulnes, Ana I. Rey, Clemente J. López-Bote, María Muñoz, Cristina Óvilo

**Affiliations:** 1 Departamento Producción Animal, Facultad de Veterinaria, Universidad Complutense de Madrid, Madrid, Spain; 2 Instituto Regional de Investigación y Desarrollo Agroalimentario y Forestal de Castilla-La Mancha (IRIAF), Toledo, Spain; 3 Departamento de Mejora Genética Animal, Instituto Nacional de Investigación y Tecnología Agraria y Alimentaria, INIA, CSIC, Madrid, Spain; 4 Centro Nacional de I+D del Cerdo Ibérico, INIA, CSIC, Zafra, Spain; 5 Sánchez Romero Carvajal S.A., Jabugo, Huelva, Spain; 6 Micros Veterinaria S.L., INDEGSAL, León, Spain; 7 Departamento de Producción y Sanidad Animal, Facultad de Veterinaria, Universidad Cardenal Herrera-CEU, Valencia, Spain; Rutgers: Rutgers The State University of New Jersey, UNITED STATES OF AMERICA

## Abstract

Antioxidant supplementation in critical periods may be useful for improvement of piglet early viability and development. We have evaluated the effects of maternal perinatal diet inclusion of a high vitamin E level (VE, 100 mg all-rac-α-tocopheryl acetate /kg), hydroxytyrosol (HT, 1.5 mg/kg), or their combination (VEHT), in comparison to a control diet (C, 30 mg all-rac-α-tocopheryl acetate /kg), on the offspring homeostasis and metabolism, analysing the weaned piglets’ adipose tissue transcriptome and adipocyte morphology. Diets were provided to pregnant Iberian sows (n = 48, 12 per treatment) from gestation day 85 to weaning (28 days postpartum) and 48 piglets (n = 12 per treatment) were sampled 5 days postweaning for dorsal subcutaneous adipose tissue analyses. RNA obtained from 6 animals for each diet was used for paired-end RNA sequencing. Results show that supplementation of sows’ diet with either vitamin E or hydroxytyrosol had substantial effects on weaned piglet adipose transcriptome, with 664 and 587 genes being differentially expressed, in comparison to C, respectively (q-value<0.10, Fold Change>1.5). Genes upregulated in C were mainly involved in inflammatory and immune response, as well as oxidative stress, and relevant canonical pathways and upstream regulators involved in these processes were predicted as activated, such as TNF, IFNB or NFKB. Vitamin E, when supplemented alone at high dose, activated lipid biosynthesis functions, pathways and regulators, this finding being accompanied by increased adipocyte size. Results suggest an improved metabolic and antioxidant status of adipose tissue in animals born from sows supplemented with individual antioxidants, while the combined supplementation barely affected gene expression, with VEHT showing a prooxidant/proinflamatory functional profile similar to C animals. Different hypothesis are proposed to explain this unexpected result. Findings allow a deeper understanding of the processes taking place in adipose tissue of genetically fat animals and the role of antioxidants in the regulation of fat cells function.

## Introduction

The perinatal period is a critical phase in the development of all mammal species and any alteration in homeostasis may affect early survival and have long lasting consequences on health and metabolism [1]. In this stage there is an unbalance in the redox potential that has been described in several species including pigs [2]. Foetal weight gain and organ maturation mainly occurs at the end of pregnancy, and the severe metabolic burden during this stage may eventually lead to sows’ systemic oxidative stress. Thus, large amounts of free radicals are produced during the birth process, when the antioxidant system of newborn piglets is weak, unable to remove excessive free radicals in time, resulting in aggravated oxidative stress [3].

Furthermore, intrauterine growth retardation (IUGR) exacerbates oxidative stress in the fetus and contributes to postnatal maladaptation of the newborn [4]. Thus, problems related to perinatal oxidative stress have been the focus of studies in hyperprolific sows with high incidence of IUGR [5], in which an increased systemic oxidative stress during late gestation and lactation has been reported.

The Iberian pig is a rustic and scarcely selected breed characterised by limited productive and reproductive efficiency and a high trend for fat deposition. Actually, this pig breed has been suggested as animal model for nutrition-associated diseases due to their susceptibility to central obesity, irregularities in insulin-glucose regulation, dyslipidemia, and high blood pressure, all indicative of metabolic syndrome [6]. This adipogenic nature has been related to the adaptation to harsh local environmental conditions by development of a thrifty, leptin resistant genotype [7]. In spite of their low prolificacy, incidence of IUGR is relevant in Iberian pigs because of maternal metabolic features and limited uterine size [8] and because of a trend for implementing maternal feed restriction along gestation for avoiding fattening and lowering production costs [9]. Thus, oxidative stress may have a major impact on early development in Iberian pigs.

Nutrition can be used as a tool to improve pig performance and health, balance litter heterogeneity-related problems, or increase animal welfare. In particular, antioxidant supplementation in critical reproductive periods may improve the offspring’s viability and development, especially in the case of IUGR [10]. A number of antioxidants, such as vitamins, minerals, probiotics and plant derived bioactive compounds (mainly fibers and plant-derived polyphenols, such as hydroxytyrosol) have been shown to alleviate oxidative stress in sows in late pregnancy and lactation stages [2], and in some cases are associated with an improvement of survival rates and birth weight [11]. In previous studies, we demonstrated perinatal antioxidant supplementation of sows had positive effects on colostrum and milk composition and stability [12], gut health [13] and early development (birth weight and body measurements) and oxidative stability of piglets at weaning [14], without significant effects on sows’ performance.

Adipose tissue in pig, with liver and muscle, are the main regulators of lipid metabolism [15]. Besides, adipose tissue acts as a major endocrine organ, producing adipocytokines and hormones involved in the maintenance of metabolic homeostasis [16]. In white adipose tissue of obese animals, there is an increased mitochondrial oxidative stress, increased generation of reactive oxygen species (ROS) and decreased antioxidant activity, coupled with alterations in adipokines required for insulin sensitivity. The oxidizing environment in adipocytes of obese individuals negatively impact the endocrine and metabolic function of fat cells, potentially altering energy balance and general homeostasis [17]. Specifically in Iberian pigs, a previous work has shown alterations in adipose tissue homeostasis, with signs of oxidative stress and development of low-grade inflammation, being developed in young animals [18].

Nutrigenomics aims to explore the biological mechanisms underlying specific feeding strategies [19] by analysing the influence of diet constituents on gene expression and tissue metabolism. In line with the explained background, it can be hypothesized that provision of enriched levels of antioxidants in the diet during specific periods of development could improve oxidative stability, lipid metabolism and homeostasis of adipose tissue, especially in genetically obese individuals. Besides, evaluation of the nutrigenomics effects of these dietary ingredients could help in understanding their role in modulating relevant molecular processes and biological functions. Thus, the objective of this work has been to evaluate the functional effects of different antioxidants (hydroxytyrosol, vitamin E and their combination), applied to Iberian sows’ diets during critical periods of development (the last third of gestation and lactation), on the adipose tissue transcriptome, cellularity and metabolic status of the offspring after weaning.

## Material and methods

### Ethics statement

All experimental procedures complied with the regulations of the Spanish Policy for Protection of Animals employed in Research and other scientific purposes (RD53/2013), which meets the European Union Directive 2010/63 /UE for the care and use of animals in research. The INIA Committee of Ethics in Animal Research approved the experimental procedures (report ORCEEA 2019–10).

### Animals and samples

The experiment was carried out at the animal facilities of *El Dehesón del Encinar* (Oropesa, Toledo, Spain). Forty eight pregnant Iberian sows (half primiparous and half multiparous with 4–5 parity) (107.2 ± 29.8 kg) were used that received a standard grain-based diet (g/kg: 888 dry matter, 124.6 crude protein, 29.9 fat, 49.3 fiber, 62.1 ash; and 3050 kcal/kg metabolizable energy) until gestation day 85. Then, sows were weighted and allotted to four homogeneous experimental groups (n = 12, with equal distribution of primiparous and multiparous) and started receiving four different experimental diets supplemented with different levels of added antioxidants (vitamin E or hydroxytyrosol) as follows:

- Control group (C): 30 mg of all-rac-α-tocopheryl acetate (Rovimix E-50, DSM)/kg feed. This dose was used to reach the minimum dietary vitamin E level recommended by the National Research Council (NRC) for sows [20].
- Vitamin E group (VE): 100 mg of all-rac-α-tocopheryl acetate/kg. This dose was chosen after taking into account the effective antioxidant effect observed in a previous study [21].
- Hydroxytyrosol group (HT): 30 mg all-rac-α-tocopheryl acetate/kg and 1.5 mg hydroxytyrosol/kg. Hydroxytyrosol dose was selected according to previous works in Iberian pigs showing a positive effect of this level of inclusion on sows’ diet on fetal antioxidant status and on piglet juvenile development [11, 22].
- Combined group (VEHT): 100 mg/kg of all-rac-α-tocopheryl acetate/kg and 1.5 mg hydroxytyrosol/kg feed.

Composition of experimental diets is shown in S1 Table. The experimental period extended from day 85 of gestation until weaning at day 28 of lactation. Feed was provided to fulfill daily maintenance requirements according to the NRC [20] and water was provided ad libitum. The α-tocopheryl acetate used in the diets was purchased from DSM Nutritional Products (Alcalá de Henares, Madrid, Spain) and the hydroxytyrosol extract (Olea europaea L. dry extract, N20130102) was obtained from Natac (Alcorcón, Madrid, Spain). Sows were group-housed till one week before farrowing, when they were moved to individual farrowing crates. Additional details on the experiment are reported in [12]. Five days post-weaning (at 33 days old), one male piglet per litter was selected (those with body weight close to the mean value of the litter) and euthanized (n = 12 per dietary treatment) by stunning and exsanguination in compliance with RD53/2013 standard procedures. Subcutaneous adipose tissue at the level of the last rib was sampled for gene expression and histological analyses. Piglet was considered the experimental unit in all analyses.

### Transcriptome sequencing

For the RNA-Seq study, samples of 24 animals were used (6 animals of each diet group, randomly selected). Total RNA was isolated from 50–100-mg samples of subcutaneous dorsal fat using the RiboPureTM RNA isolation kit (Ambion, Austin, TX, USA), following the manufacturer’s recommendations. The RNA was quantified using a NanoDrop device (NanoDrop Technologies, Wilmington, DE, USA), and the RNA quality was assessed with an Agilent 2100 bioanalyzer (Agilent Technologies, Palo Alto, CA, USA). RNA integrity numbers (RIN coefficient) had mean value of 7.8±0.4. Sequencing was performed in an external service (Macrogen). Libraries were prepared by using the TruSeq mRNA-Seq sample preparation kit (Illumina Inc., San Diego, CA, USA) according to the manufacturer’s protocol. Each library was paired-end sequenced (2 × 150bp) by using TruSeq SBS Kit v3-HS in a HiSeq2000 platform (Illumina, Inc.).

### RNA-Seq data analyses

The quality of raw sequences was assessed with FastQC [23]. Sequencing data was trimmed with TrimGalore (Babraham Bioinformatics, http://www.bioinformatics.babraham.ac.uk/projects/trim_galore/) with default settings to remove the sequencing adaptors and poly A and T tails, keeping only paired-end reads where both pairs were longer than 40 bp. Filtered reads were mapped against the pig reference genome (Sscrofa11.1) using HISAT [24].

Raw counts for the genes and transcripts were obtained with HTSeq-counts [25] and after, statistical analyses of differential expression were carried out using DESeq2 R package [26], setting a FDR adjusted q-value ≤ 0.10 and Fold Change (FC) ≥ 1.5. A relaxed FDR threshold was applied (10% of false positives are expected), considering the exploratory nature of the analyses, in order to retain information that could have been lost with a more restrictive approach. DESeq2 software analyses expression differences under an inverse binomial distribution assumption, presuming that the majority of genes are not differentially expressed. It calculates normalization factors based on the medians of observed count ratios. In the present study the model included the diet effect in two ways:

1.- Factorial analysis: all experimental groups were analyzed together with a factorial model, including the effects of vitamin E and hydroxytyrosol levels, as well as the interaction effect of both antioxidants.
2.-Pair-wise transcriptome comparisons: each pair of experimental groups were included and analyzed with a model including the diet effect (six transcriptome comparisons were made).

Genes with non-detected expression for ≥75% of samples were removed (≥9 samples with 0 counts).

The Database for Annotation, Visualization and Integrated Discovery (DAVID) tool [27] was used for the functional annotation of the differentially expressed genes (DEGs). DAVID was employed to explore the enrichment of biological functions and metabolic and signalling pathways in each set of DEGs, by analysing separately the genes upregulated in each one of the compared experimental groups. Besides, functional interpretation of the differential expression findings was performed with the Ingenuity Pathway Analysis (IPA) software (Ingenuity Systems, Qiagen, CA, United States) for the identification of activated and inhibited canonical pathways and potential regulators for the two main comparisons performed (C vs VE and C vs HT), as well as for the construction of functional networks. Functional analyses on IPA software were run with default settings, excepting that: “mammals” were only selected in the Species section, “cell lines” were excluded in the Tissues &cell lines section; and “interaction + causal networks” were selected in the Networks section.

Raw sequencing data files as well as processed gene expression data have been deposited in GEO database under accession number GSE249774.

### Validation by qPCR

RNA obtained from the same 24 animals used in the RNA-Seq study was employed to perform the technical validation of differential expression results by qPCR. Design of amplification primers for each gene was done using Primer Select Software (DNASTAR, Wisconsin, USA) from available ENSEMBL sequences, covering different exons to assure the amplification of the cDNA. The specific primer details are shown in S2 Table. Retrotranscription was carried out with SuperScript III (Invitrogen, Life Technologies, Paisley, UK) and random hexamers in a total volume of 20 μl containing 1 μg of total RNA, following the supplier´s instructions.

Validation was carried out for eight genes (*GPX1*, *SCD*, *JAZF1*, *NR4A3*, *LEP*, *CASP1*, *PON3 and TLR2*), using *eEF2* and *TBP* as housekeeping genes. Selection of genes for validation was done by including DE genes with a range of FC values in the different transcriptome comparisons. Besides, three out of the selected genes were chosen because of their relevant role in adipose tissue biology in Iberian pigs and antioxidant response (*SCD*, *LEP* and *GPX1*). The selection of the two most stable endogenous genes for data normalization was done by evaluating *GAPDH*, *ACTB*, *TBP*, *18S*, *PPIA*, *eEF2* and *B2M* with the Genorm software [28]. Transcripts quantification was performed using SYBR green mix (Roche, Basel, Switzerland) in a LightCycler 480 II (Roche). The method proposed by Steibel [29], was employed for the statistical analysis of qPCR gene expression data, following the procedure explained in previous works [18]. Pearson correlations between the expression values obtained from RNA-Seq data (counts) and the normalized gene expression data obtained by qPCR were used for assessing the technical validation. The p-values < 0.05 were considered statistically significant.

### Histological analyses

Adipose tissue samples were fixed in 10% neutral buffered formalin and embedded in paraffin, cut at 4 μm and stained with hematoxylin and eosin for examination. The measurement of the diameter of 30 adipocytes per sample was performed and averaged. These measurements were made with the Image J^™^ software (U.S. National Institutes of Health, Bethesda, MD, USA), on photomicrographs taken with a Leica ICC50W^™^ camera coupled to a Leica DM1000^™^ microscope (Leica, Mannheim, Germany). The influence of maternal diet on piglet’s adipocyte diameter was analyzed with a linear model fitting the diet group as fixed-effect, using the MIXED procedure of SAS 9.4 (SAS Institute Inc., Cary, NC, USA) as well as evaluating the global effects of vitamin E (30 vs 100mg/kg) and hydroxytyrosol (0 vs 1.5mg/kg) supplementation. The results were considered to be significant at p-value < 0.05 and tendency was considered for p-value<0.10.

## Results

### Differential expression and functional interpretation

RNA-Seq was used to profile the adipose tissue transcriptome of 24 Iberian piglets, in order to study the effect of sows’ diets supplemented with different antioxidant agents. An average of 64 million sequence reads were obtained for each sample, and were assembled and mapped to the annotated Sscrofa11.1 genome. All samples successfully passed the quality control, with 96% of the reads mapped to the porcine reference sequence. An average of 14,300 genes out of the 22,452 annotated genes were expressed in the studied samples. A figure illustrating Principal Component Analysis of the 24 samples according to transcriptome data is available as S1 Fig.

Differential gene expression analysis from transcriptome data was done by applying different models:

When a factorial model was applied, including both the vitamin E and the hydroxytyrosol effects and the interaction effect, only 4 genes were influenced by the vitamin E level and 1 was affected by hydroxytyrosol level (q<0.10 and FC ≥ 1.5). DEGs influenced by vitamin E were *SLC30A2*, *MDFI*, *and SNORD123*, upregulated in the adipose tissue of piglets born to sows supplemented with 100 mg of all-rac-α-tocopheryl acetate/kg (VE and VEHT groups)*; and C17orf98*, upregulated in those born to sows receiving the basal vitamin E level (C and HT groups). *SERPINB11* was the only DEG affected by hydroxytyrosol level after the application of a factorial model, being upregulated in animals born to mothers non-supplemented with hydroxytyrosol (C and VE groups). In contrast to the scarce effects of the two supplementations applied, the interaction vitamin E * hydroxytyrosol effect was significant for 932 genes, included in S3 Table (q<0.10). Two main types of interactions were observed. In first place 83 qualitative interactions were detected, in which the effect of one antioxidant was reversed when the other antioxidant was added. We also observed a high number of quantitative interactions (n = 590), for which the magnitude or significance of the effects varied depending on the supplementation with only one or both antioxidants. Most of these quantitative interactions meant that the effect on gene expression was only observed with statistical significance after the individual supplementation of one of the two evaluated antioxidants.

**Fig 1:**
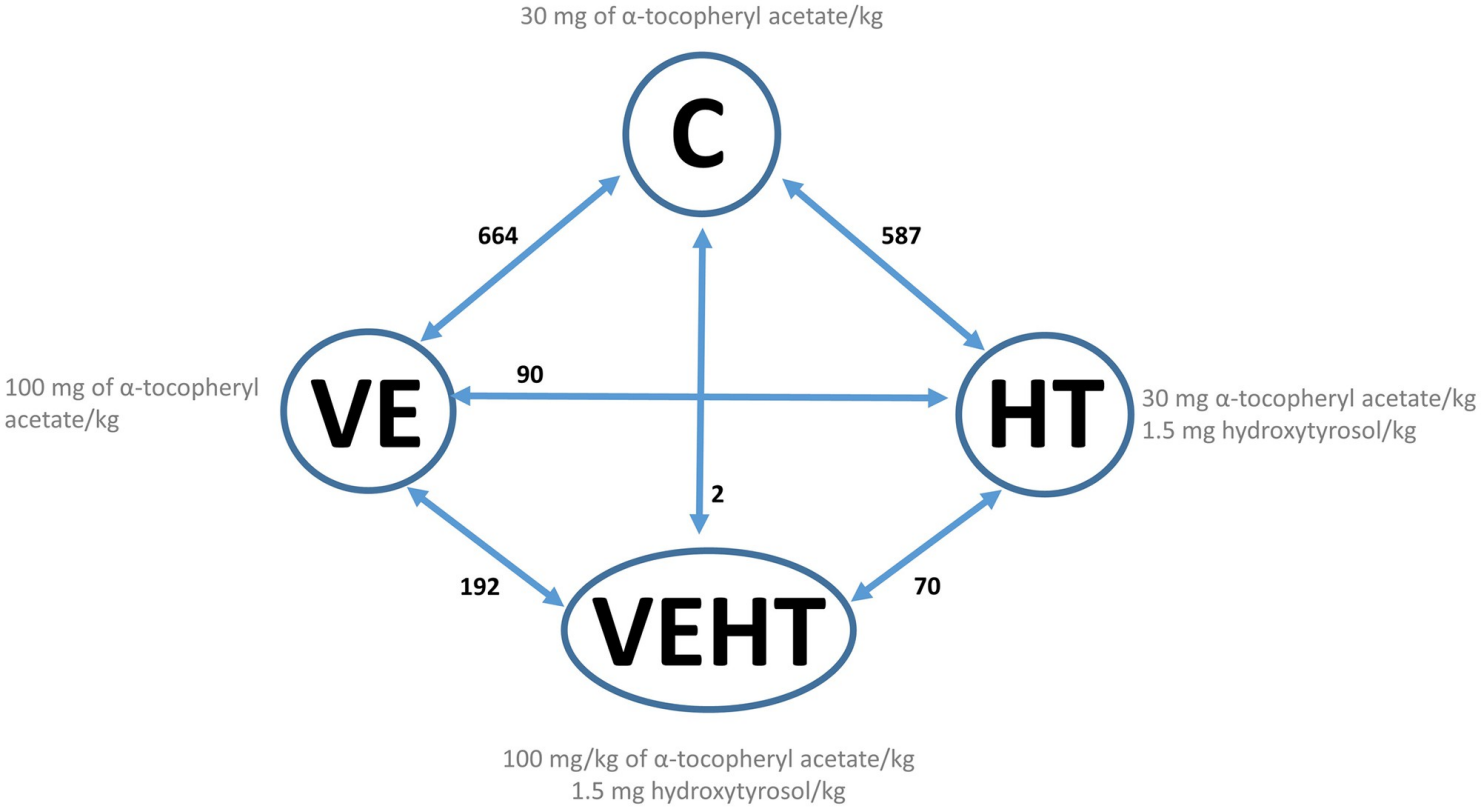
Transcriptome comparisons and differential expression. Numbers beside each arrow indicate the number of DEGs in each comparison (q<0.10 and FC ≥ 1.5).

Due to the complexity of interpretation of interaction effects, six different pair-wise transcriptome contrasts were also performed among the four experimental groups. Comparisons made and number of DEGs detected in each of them is shown in Fig 1. The lists of DEGs corresponding to each contrast are available in S4 Table (q<0.10 and FC ≥ 1.5).

**Fig 2:**
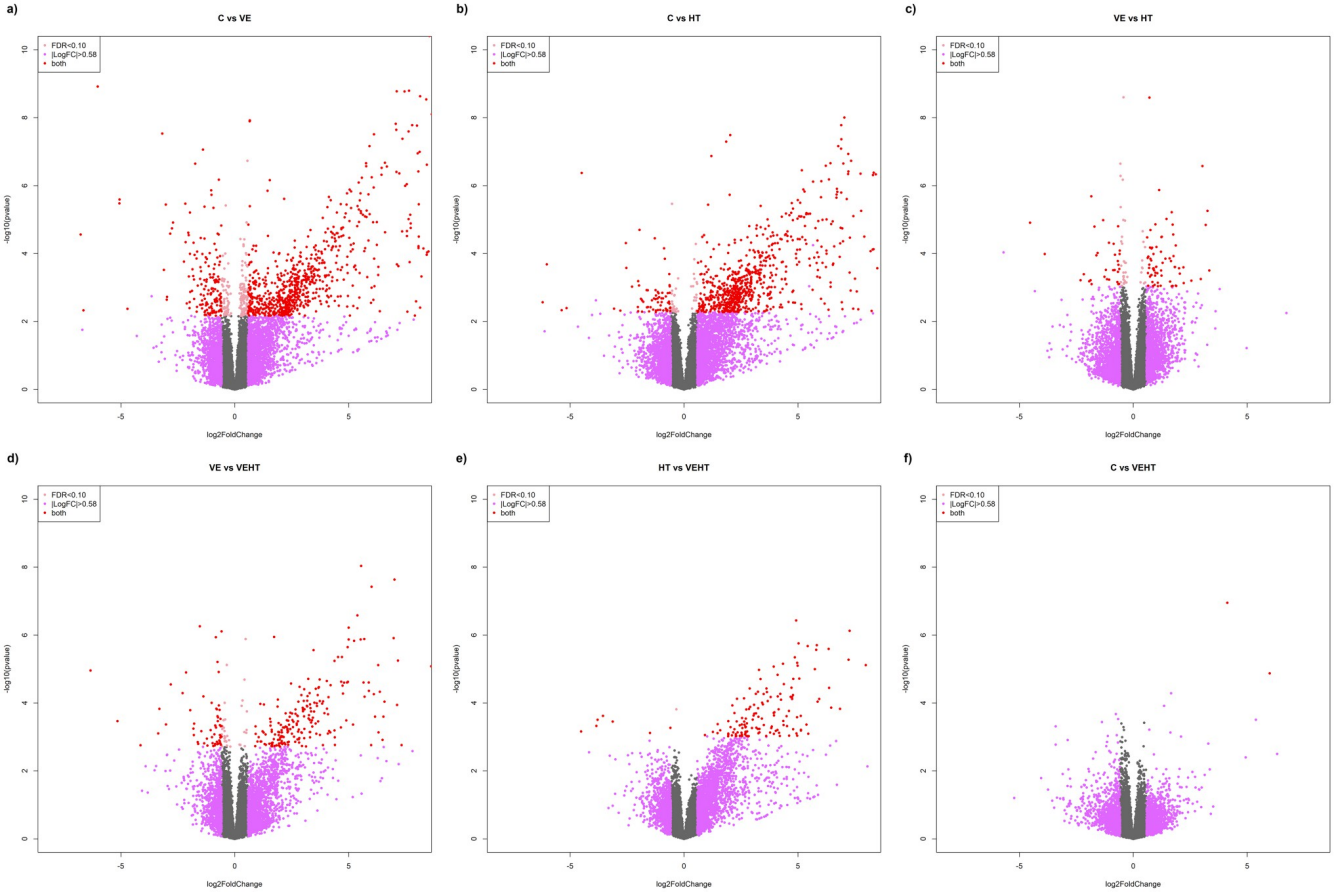
Volcano plots corresponding to the six transcriptome comparisons performed among experimental groups (q<0.10 and FC ≥ 1.5). Plots show fold-change and p-value for each gene in the different comparisons. Differentially expressed genes are depicted as red dots.

The diets that exhibited the most significant impact on adipose tissue transcriptome were those supplemented with vitamin E, followed by hydroxytyrosol supplemented ones (both supplemented individually), when compared to the control group. Both showed similar number of DEGs (664 and 587, respectively). In contrast, a more limited number of DEGs were observed when comparing these two treatments to each other (VE vs HT; n = 90 DEGs), and negligible results were found for the comparison of the combined treatment (VEHT) vs the control group (2 DEGs). When we analyzed the effect of hydroxytyrosol supplementation under a high level of vitamin E (VE vs VEHT), and the effect of vitamin E in diets supplemented with hydroxytyrosol (HT vs VEHT), 192 and 70 DEGs were found, respectively. Volcano plots corresponding to the six transcriptome comparisons are included in Fig 2.

#### Transcriptome comparison VE vs C

Vitamin E, when supplemented alone, induced the change of expression of 664 genes, with 527 DEGs genes being upregulated in C and 137 in VE (S4 Table). The genes with the highest fold change values within each group were *HPS5* (489x), upregulated in C, and *JAKMIP2* (64x), upregulated in VE.

Functional enrichment analysis results are shown in S5 Table. Genes upregulated in C group were involved in *inflammatory response* (p_adj_ = 1.6x10^-21^) and *immune response* (p_adj_ = 1.4x10^-19^), *cytokine interactions* (p_adj_ = 1.6x10^-18^), *chemokine signalling* (p_adj_ = 1.3x10^-13^) and many other biological processes related to the development of inflammation. Also, processes related to generation of oxygen free radicals and oxidative stress were enriched in C group, such as *positive regulation of superoxide anion generation* (p_adj_ = 4.4x10^-3^). Genes upregulated in VE were mainly involved in *immunity* (p_adj_ = 1.1x10^-2^) and *metabolic pathways* (p_adj_ = 9.5x10^-3^), with *lipid metabolism* and *lipid biosynthetic processes* being significantly enriched (p_adj_ = 1.6x10^-2^ and 2.7x10^-2^, respectively). Interestingly, the term *immunity* was enriched in both the C and the VE groups. Enrichment of this term in C was associated to differential expression of 41 genes (q value = 5x10^-19^), mainly including genes coding for proinflammatory molecules, while enrichment of this same *immunity* term in VE was associated to differential expression of ten genes (q value = 0.01), which were mostly genes coding for collagens.

Canonical pathways were predicted as activated or inhibited as a consequence of the differential expression by using IPA software. Results are included in S6 Table and a summary of the most interesting results for the comparison between C and VE groups are shown in Table 1. Results agree with the functional enrichment findings, with many different canonical pathways involved in immune and inflammatory responses being predicted as activated in C group. Besides, two key pathways related to oxidative stress were activated in the C group: *Production of Nitric Oxide and Reactive Oxygen Species* (p_adj_ = 1x10^-10^) and *iNOS Signaling* (p_adj_ = 4x10^-7^).

**Table 1:** Most relevant canonical pathways detected as activated or inhibited in the contrast C vs VE.

Canonical Pathways	p-value	z score	Molecules
**Activated in VE**			
*Antioxidant Action of Vitamin C*	4.5x10^-7^	-3.464	*CSF2*,*CSF2RB*,*IKBKE*,*NFKB2*,*NFKBIE*,*PLA2G2D*,*PLA2G2F*,*PLA2G5*,*PLA2G7*,*PLCB2*,*PLCG2*,*RELB*,*SLC23A1*,*SLC2A5*,*TNF*
*LXR/RXR Activation*	1x10^-8^	-2.496	*ACACA*,*C3*,*CD14*,*IL18RAP*,*IL1A*,*IL1RL2*,*LPL*,*LYZ*,*NFKB2*,*NGFR*,*PON3*,*PTGS2*,*RBP4*,*RELB*,*S100A8*,*SCD*,*SREBF1*,*TNF*
*PPAR Signaling*	0.0001	-1.897	*IKBKE*,*IL18RAP*,*IL1A*,*IL1RL2*,*MRAS*,*NFKB2*,*NFKBIE*,*NGFR*,*PTGS2*,*RELB*,*TNF*
*Erythropoietin Signaling*	3.8x10^-9^	-1.528	*CD40LG*,*CSF2*,*CSF2RB*,*CXCL8*,*IFNG*,*IL1A*,*LEP*,*LTA*,*MRAS*,*NFKB2*,*NFKBIE*,*PIK3CD*,*PIK3R1*,*PIK3R5*,*PRKCB*,*RAC2*,*RELB*,*TGFB1*,*TNF*,*TNFSF13B*,*TNFSF14*,*TNFSF15*
*PTEN Signaling*	1.7x10^-7^	-1.508	*CBL*,*IGF1R*,*IGF2R*,*IKBKE*,*INPP5F*,*ITGAL*,*ITGAM*,*ITGB2*,*ITGB7*,*MRAS*,*NFKB2*,*NGFR*,*NTRK1*,*PIK3CD*,*PIK3R1*,*PIK3R5*,*RAC2*,*RELB*
*PPARα/RXRα Activation*	0.0030	-1.000	*ADCY8*,*IKBKE*,*IL18RAP*,*IL1RL2*,*LPL*,*MRAS*,*NFKB2*,*NFKBIE*,*PLCB2*,*PLCG2*,*PRKCB*,*RELB*,*TGFB1*
*Triacylglycerol Biosynthesis*	0.0128	-1.000	*ASPG*,*DGAT2*,*ELOVL6*,*LPCAT2*,*PLPP2*
**Activated in C**			
*Phagosome Formation*	4x10^-15^	6.971	*ADGRE3*,*ADGRG5*,*ADORA3*,*BDKRB1*,*C3*,*C5AR1*,*CCR1*,*CCR2*,*CCR4*,*CCR5*,*CCR7*,*CCR8*,*CCRL2*,*CD14*,*CLEC7A*,*CNR2*,*CXCR6*,*DIAPH1*,*FCGR1A*,*FFAR2*,*FFAR4*,*FGR*,*GABBR2*,*GPR160*,*GPR171*,*GPR174*,*GPR18*,*GPR35*,*GPR82*,*HCK*,*HRH2*,*ITGAL*,*ITGAM*,*ITGB2*,*ITGB7*,*LCK*,*MARCO*,*MRAS*,*MRC1*,*P2RY10*,*P2RY11*,*P2RY13*,*P2RY6*,*PIK3CD*,*PIK3R1*,*PIK3R5*,*PLA2G2D*,*PLA2G2F*,*PLA2G5*,*PLA2G7*,*PLCG2*,*PRKCB*,*PTAFR*,*RAC2*,*RASGRP1*,*SYK*,*TLR2*,*TLR8*,*VAV3*,*XCR1*
*FAK Signaling*	1x10^-10^	5.986	*ADGRE3*,*ADGRG5*,*ADORA3*,*BDKRB1*,*C5AR1*,*CAPN8*,*CCR1*,*CCR2*,*CCR4*,*CCR5*,*CCR7*,*CCR8*,*CCRL2*,*CD3D*,*CD3E*,*CD3G*,*CNR2*,*COL1A2*,*COL3A1*,*COL5A3*,*CSF2RB*,*CXCR6*,*ECM2*,*ETS2*,*FFAR2*,*FFAR4*,*GABBR2*,*GPR160*,*GPR171*,*GPR174*,*GPR18*,*GPR35*,*GPR82*,*HRH2*,*IFNAR1*,*IGF1R*,*IL10RA*,*IL12RB1*,*IL18RAP*,*IL1RL2*,*IL21R*,*IL22RA2*,*IL7R*,*ITGAL*,*ITGAM*,*ITGB2*,*ITGB7*,*LCK*,*LEF1*,*MRAS*,*NFKB2*,*P2RY10*,*P2RY11*,*P2RY13*,*P2RY6*,*PIK3CD*,*PIK3R1*,*PIK3R5*,*PLCG2*,*PTAFR*,*SH2D2A*,*SOCS3*,*SP1*,*TCF7*,*TGFB1*,*TRDC*,*XCR1*
*CREB Signaling*	6x10^-10^	5.308	*ADCY8*,*ADGRE3*,*ADGRG5*,*ADORA3*,*BDKRB1*,*C5AR1*,*CCR1*,*CCR2*,*CCR4*,*CCR5*,*CCR7*,*CCR8*,*CCRL2*,*CNR2*,*CREB3L3*,*CXCR6*,*FFAR2*,*FFAR4*,*GABBR2*,*GPR160*,*GPR171*,*GPR174*,*GPR18*,*GPR35*,*GPR82*,*GRIA2*,*HRH2*,*IGF1R*,*IGF2R*,*MRAS*,*NGFR*,*NTRK1*,*P2RY10*,*P2RY11*,*P2RY13*,*P2RY6*,*PIK3CD*,*PIK3R1*,*PIK3R5*,*PLCB2*,*PLCG2*,*PRKCB*,*PTAFR*,*RPS6KA1*,*TGFB1*,*XCR1*
*T Cell Receptor Signaling*	5x10^-8^	4.218	*B2M*,*CARD11*,*CBL*,*CD28*,*CD3D*,*CD3E*,*CD3G*,*CD4*,*CD80*,*CD8B*,*CSF2*,*DUSP5*,*FYB1*,*HLA-DRA*,*ICAM1*,*ICOS*,*IFNG*,*IKBKE*,*ITGAL*,*ITGB2*, *LCK*,*LCP2*, *LEF1*,*MRAS*,*NFKB2*,*NFKBIE*,*PIK3CD*,*PIK3R1*,*PIK3R5*,*PTPN22*,*PTPN7*,*PTPRC*,*RASGRP1*,*RELB*,*SKAP1*,*TCF7*,*TNF*,*TRDC*,*VAV3*,*ZAP70*
*Natural Killer Cell Signaling*	1x10^-15^	4.131	*B2M*,*CD226*,*CD244*,*CD48*,*COL1A2*,*COL3A1*,*COL5A3*,*IFNG*,*IL12RB1*,*IL18RAP*,*ITGAL*,*JAK3*,*KLRC4-KLRK1/KLRK1*,*LAIR1*,*LCK*,*LCP2*, *MAP3K8*, *MRAS*,*NCR1*,*NFKB2*,*PIK3CD*,*PIK3R1*,*PIK3R5*,*PLCG2*,*RAC2*,*RELB*,*SH2D1A*,*STAT4*,*SYK*,*ULBP1*,*VAV3*,*ZAP70*
*TREM1 Signaling*	2x10^-10^	3.873	*CASP1*,*CD40*,*CIITA*,*CSF2*,*CXCL8*,*ICAM1*,*NFKB2*,*NLRC3*,*NLRP12*,*NLRP3*,*PLCG2*,*RELB*,*TLR2*,*TLR8*,*TNF*,*TREM1*
*Th1 Pathway*	2x10^-20^	3.674	*CCR5*,*CD274*,*CD28*,*CD3D*,*CD3E*,*CD3G*,*CD4*,*CD40*,*CD40LG*,*CD80*,*CXCR3*,*HAVCR2*,*HLA-DRA*,*ICAM1*,*ICOS*, *IFNAR1*,*IFNG*,*IL10RA*, *IL12RB1*, *IL27*,*IRF1*,*ITGB2*,*JAK3*,*LTA*,*PIK3CD*,*PIK3R1*,*PIK3R5*,*RUNX3*,*SOCS3*,*STAT4*
*p38 MAPK Signaling*	2x10^-5^	3.464	*CREB3L3*,*IL18RAP*,*IL1A*,*IL1RL2*,*IRAK2*,*IRAK3*,*MAP4K1*,*PLA2G2D*,*PLA2G2F*,*PLA2G5*,*RPS6KA1*,*TGFB1*,*TNF*
*PI3K Signaling*	7x10^-12^	3.411	*ATF3*,*BLK*,*C3*,*CBL*,*CD180*,*CD19*,*CD40*,*CD79A*,*DAPP1*,*IKBKE*,*MRAS*,*NFKB2*,*NFKBIE*,*PIK3AP1*,*PIK3CD*,*PIK3R1*,*PLCB2*,*PLCG2*,*PRKCB*,*PTPRC*,*RELB*,*SYK*,*VAV3*
*Production of Nitric Oxide and ROS*	1x10^-10^	3.411	*IFNG*,*IKBKE*,*IRF1*,*IRF8*,*JAK3*,*LYZ*,*MAP3K8*,*NCF1*,*NFKB2*,*NFKBIE*,*NGFR*,*PIK3CD*,*PIK3R1*,*PIK3R5*,*PLCG2*,*PRKCB*,*RAC2*,*RBP4*,*RELB*,*RHOH*,*RND1*,*RND2*,*S100A8*,*TLR2*,*TNF*
*IL-8 Signaling*	9x10^-8^	3.300	*CXCL8*,*HBEGF*,*ICAM1*,*IKBKE*,*IRAK2*,*IRAK3*,*ITGAM*,*ITGB2*,*MRAS*,*NCF1*,*NFKBIE*,*PIK3CD*,*PIK3R1*,*PIK3R5*,*PLCB2*,*PRKCB*,*PTGS2*,*RAC2*,*RHOH*,*RND1*,*RND2*,*VASP*
*NF-κB Signaling*	0.0002	3.272	*CARD11*,*CD3D*,*CD3E*,*CD3G*,*CD40*,*CD40LG*,*IGF1R*,*IGF2R*,*IL1A*,*IRAK3*,*LCK*,*LTA*,*MAP3K8*,*MRAS*,*NFKB2*,*NFKBIE*,*NGFR*,*NTRK1*,*PIK3CD*,*PIK3R1*,*PIK3R5*,*PLCG2*,*PRKCB*,*RELB*,*TLR2*,*TLR8*,*TNF*,*TNFRSF17*,*TNFSF13B*,*TRDC*,*ZAP70*
*Insulin Secretion Signaling*	0.0089	3.207	*ADCY8*,*CREB3L3*,*FGR*,*HCK*,*JAK3*,*LCK*,*PIK3CD*,*PIK3R1*,*PIK3R5*,*PLCB2*,*PLCG2*,*PRKCB*,*SLC2A5*,*STAT4*,*STX1B*
*iNOS Signaling*	4x10^-7^	2.828	*CD14*,*IFNG*,*IKBKE*,*IRAK2*,*IRAK3*,*IRF1*,*JAK3*,*NFKB2*,*NFKBIE*,*RELB*
*ERK/MAPK Signaling*	2x10^-6^	2.000	*CREB3L3*,*DUSP2*,*ESR1*,*ETS2*,*ITGAL*,*ITGAM*,*ITGB2*,*ITGB7*,*KSR1*,*MRAS*,*PIK3CD*,*PIK3R1*,*PIK3R5*,*PLA2G2D*,*PLA2G2F*,*PLA2G5*,*PLCG2*,*PRKCB*,*RAC2*,*RPS6KA1*

Positive z scores indicate activation in C, negative ones indicate activation in VE. C: 30 mg of α-tocopheryl acetate/kg feed; VE: 100 mg of α-tocopheryl acetate/kg.

**Fig 3:**
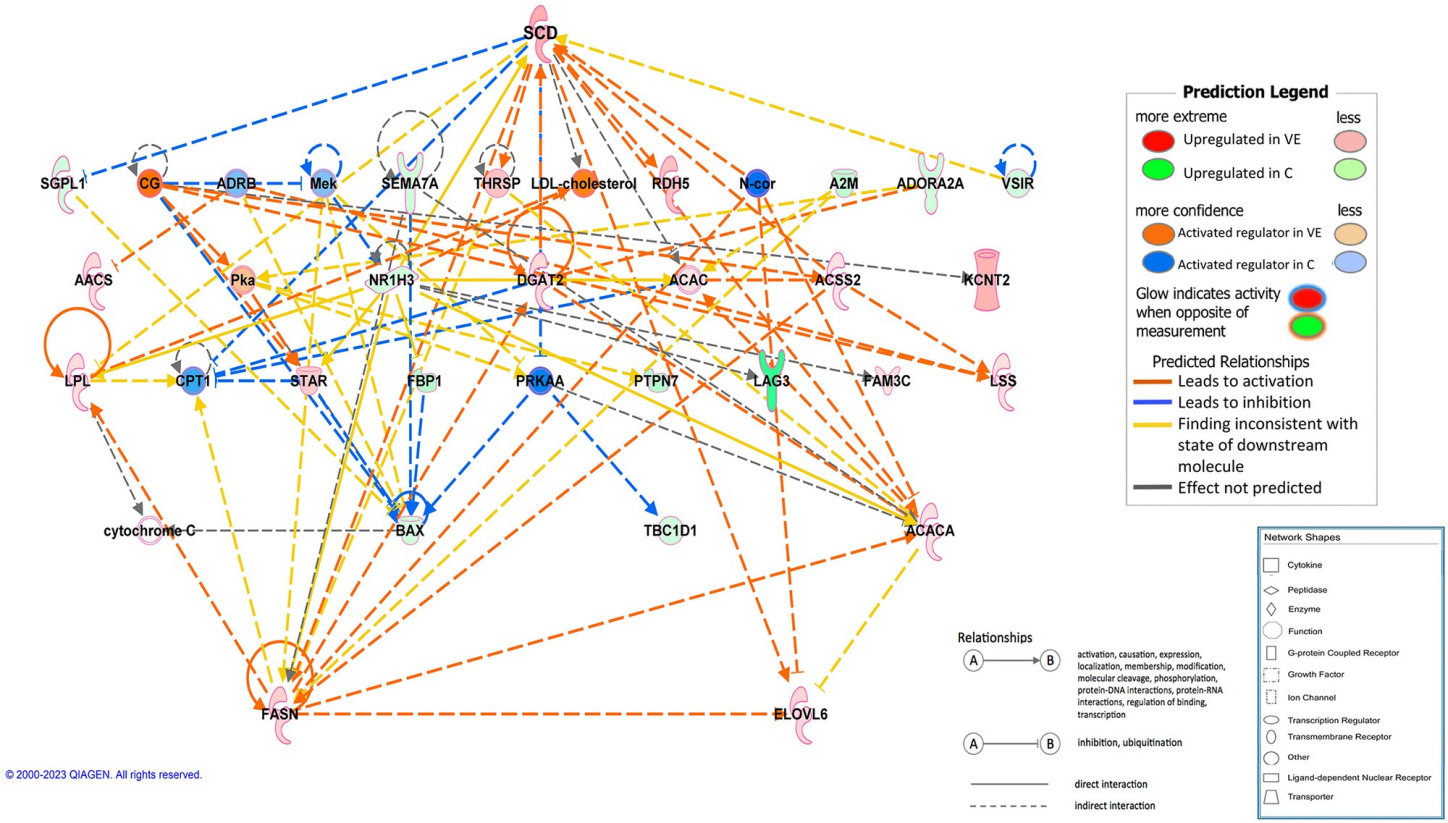
Functional gene network predicted by IPA and involving genes related to lipid sinthesis and metabolism, upregulated in VE group in comparison to C group. Orange nodes indicate molecules upregulated in VE. Green nodes indicate molecules upregulated in C.

On the contrary, in the VE group, most activated canonical pathway was *Antioxidant action of vitamin C* (p_adj_ = 4x10^-7^), followed by several ones involved in lipid metabolic processes, such as *LXR*, *RXR*, *PPAR signalling* or *triacylglycerol synthesis pathways*, as well as *erythropoietin signalling* and *PTEN signalling*. A functional network was predicted by IPA involving the DEGs related to lipid biosynthetic processes and is shown in Fig 3, where *SCD* gene has a central role.

#### Transcriptome comparison HT vs C

Hydroxytyrosol individual supplementation led to differential expression of 587 genes, 531 being upregulated in the C group and 56 being upregulated in HT (S4 Table). Genes with the highest FC values were those with increased expression in C, such as *CCL1* (634x) or *CCL22* (314x). *SBK2* was the DEG upregulated in HT with highest expression difference (65x). Functional enrichment (S5 Table) of the set of genes upregulated in C showed similar results to those observed in the previous contrast, with overrepresentation of functional terms and pathways involved in *inflammatory response* (p_adj_ = 8.8x10^-19^) and *immune response* (p_adj_ = 9.4x10^-11^), *cytokine interactions* (p_adj_ = 3.2x10^-13^), *chemokine signalling* (p_adj_ = 3.2x10^-11^), *interleukin production* (p_adj_ = 2x10^-10^) and many other immune and signalling processes. Again, oxidative stress related functions were found potentially activated in C group, as *positive regulation of superoxide anion generation* (p_adj_ = 2.9x10^-3^) or *superoxide-generating NADPH oxidase activity* (p_adj_ = 4.3x10^-3^). The only term significantly enriched in the genes upregulated in HT was *oxidoreductase activity* (p_adj_ = 0.05).

Canonical pathways potentially activated as a consequence of the DEGs are listed in S6 Table and a summary of the most relevant results for the comparison between C and HT groups are shown in Table 2. Results agree with the functional enrichment findings, and are, once again, similar to C vs VE transcriptome comparison. Canonical pathways activated in the C group showed induction of a large number of canonical pathways involved in immune and inflammatory responses as well as key pathways related to oxidative stress: *Production of Nitric Oxide and Reactive Oxygen Species* (p_adj_ = 1x10^-10^) and *iNOS Signaling* (p_adj_ = 0.005). In the HT group, a slightly lower number of activated canonical pathway was observed, in comparison to the findings in the VE contrast. Activated pathways were similar to those induced by vitamin E, including: *Antioxidant action of vitamin C*, *LXR/RXR Activation*, *PPAR Signaling* and *Erythropoietin Signaling*.

**Table 2:** Most relevant canonical pathways detected as activated or inhibited in the contrast C vs HT.

Canonical Pathways	p-value	z score	Molecules
**Activated in HT**			
*Antioxidant Action of Vitamin C*	9x10^-8^	-3.464	*ABHD3*,*CSF2*,*CSF2RB*,*IKBKE*,*NAPEPLD*,*PLA2G2D*,*PLA2G2F*,*PLA2G5*,*PLA2G7*,*PLCB2*,*PLCG2*,*SLC23A1*,*SLC23A2*,*SLC2A5*,*TNF*
*LXR/RXR Activation*	2x10^-6^	-2.673	*ARG2*,*C3*,*CCL2*,*CD14*,*IL18*,*IL1A*,*IL1R2*,*KNG1*,*LBP*,*LYZ*,*MMP9*,*S100A8*,*TLR4*,*TNF*
*PPAR Signaling*	0.0467	-1.633	*IKBKE*,*IL18*,*IL1A*,*IL1R2*,*PDGFD*,*TNF*
*Erythropoietin Signaling*	0.0001	-1.604	*CSF2*,*CSF2RB*,*CXCL8*,*IL18*,*IL1A*,*PIK3CD*,*PIK3R1*,*PIK3R5*,*PRKCD*,*PTPN6*,*RAC3*,*TNF*,*TNFSF13B*,*TNFSF15*
**Activated in C**			
*Phagosome Formation*	5x10^-14^	6.532	*ABHD3*,*ADGRE3*,*ADGRV1*,*AP1S2*,*AP1S3*,*C3*,*C5AR1*,*CCR1*,*CCR2*,*CCR4*,*CCR5*,*CCR7*,*CCRL2*,*CD14*,*CLEC7A*,*CMKLR1*,*CNR2*,*CXCR6*,*FFAR2*,*FGR*,*GPR160*,*GPR171*,*GPR35*,*GRM7*,*HCK*,*HRH2*,*ITGAM*,*ITGB2*,*LBP*,*LTB4R*,*MARCO*,*MRC1*,*NAPEPLD*,*P2RY11*,*P2RY13*,*P2RY6*,*PIK3CD*,*PIK3R1*,*PIK3R5*,*PLA2G2D*,*PLA2G2F*,*PLA2G5*,*PLA2G7*,*PLCG2*,*PRKCD*,*PTAFR*,*RAC3*,*SYK*,*TLR2*,*TLR4*,*TLR6*,*TLR8*,*VAV1*,*XCR1*
*CREB Signaling*	6x10^-6^	5.396	*ADCY7*,*ADGRE3*,*ADGRV1*,*C5AR1*,*CCR1*,*CCR2*,*CCR4*,*CCR5*,*CCR7*,*CCRL2*,*CMKLR1*,*CNR2*,*CXCR6*,*FFAR2*,*GPR160*,*GPR171*,*GPR35*,*GRM7*,*HRH2*,*LTB4R*,*NTRK1*,*P2RY11*,*P2RY13*,*P2RY6*,*PIK3CD*,*PIK3R1*,*PIK3R5*,*PLCB2*,*PLCG2*,*POLR2I*,*PRKCD*,*PTAFR*,*RPS6KA1*,*XCR1*
*FAK Signaling*	0.0009	5.246	*ADGRE3*,*ADGRV1*,*C5AR1*,*CCR1*,*CCR2*,*CCR4*,*CCR5*,*CCR7*,*CCRL2*,*CDH1*,*CMKLR1*,*CNR2*,*CSF2RB*,*CXCR6*,*FFAR2*,*GPR160*,*GPR171*,*GPR35*,*GRM7*,*HRH2*,*IFNAR1*,*IL10RA*,*IL1R2*,*IL21R*,*IL27RA*,*IL7R*,*ITGAM*,*ITGB2*,*LTB4R*,*MMP9*,*P2RY11*,*P2RY13*,*P2RY6*,*PIK3CD*,*PIK3R1*,*PIK3R5*,*PLCG2*,*PTAFR*,*SOCS3*,*TCF7*,*TRDC*,*XCR1*
*TREM1 Signaling*	1x10^-15^	4.472	*CASP1*,*CCL2*,*CD40*,*CD86*,*CSF2*,*CXCL8*,*IL18*,*LAT2*,*NLRP12*,*NLRP3*,*NOD1*,*NOD2*,*PLCG2*,*TLR2*,*TLR4*,*TLR6*,*TLR8*,*TNF*,*TREM1*,*TYROBP*
*Production of Nitric Oxide and ROS in Macrophages*	3x10^-10^	3.578	*ARG2*,*CYBA*,*IKBKE*,*IRF8*,*JAK3*,*LYZ*,*MAP3K9*,*NCF1*,*NCF2*,*NCF4*,*PIK3CD*,*PIK3R1*,*PIK3R5*,*PLCG2*,*PRKCD*,*PTPN6*,*RAC3*,*RND2*,*S100A8*,*SPI1*,*TLR2*,*TLR4*,*TNF*
*IL-17 Signaling*	0.0002	3.207	*CCL17*,*CCL2*,*CCL22*,*CSF2*,*CXCL8*,*IL18*,*IL1A*,*MMP9*,*PIK3CD*,*PIK3R1*,*PIK3R5*,*TNF*,*TNFSF13B*,*TNFSF15*
*PI3K Signaling*	1x10^-8^	3.153	*BLK*,*BLNK*,*BTK*,*C3*,*CBL*,*CD180*,*CD40*,*CD79B*,*DAPP1*,*IKBKE*,*PIK3AP1*,*PIK3CD*,*PIK3R1*,*PLCB2*,*PLCG2*,*SYK*,*TLR4*,*VAV1*
*IL-15 Production*	0.0031	3.000	*ABL2*,*BLK*,*BTK*,*FGR*,*HCK*,*JAK3*,*NTRK1*,*SYK*,*TNK1*
*Inflammasome pathway*	1x10^-8^	2.828	*CASP1*,*CTSB*,*IL18*,*NLRP3*,*NOD2*,*PANX1*,*PYCARD*,*TLR4*
*IL-8 Signaling*	6x10^-5^	2.673	*CDH1*,*CXCL8*,*IKBKE*,*ITGAM*,*ITGB2*,*MMP9*,*NAPEPLD*,*NCF1*,*NCF2*,*PIK3CD*,*PIK3R1*,*PIK3R5*,*PLCB2*,*PRKCD*,*RAC3*,*RND2*
*p38 MAPK Signaling*	0.0091	2.646	*IL18*,*IL1A*,*IL1R2*,*PLA2G2D*,*PLA2G2F*,*PLA2G5*,*RPS6KA1*,*TNF*
*Th1 Pathway*	4x10^-8^	2.496	*CCR5*,*CD28*,*CD40*,*CD86*,*IFNAR1*,*IL10RA*,*IL18*,*IL27*,*IL27RA*,*ITGB2*,*JAK3*,*PIK3CD*,*PIK3R1*,*PIK3R5*,*SOCS3*,*VAV1*
*HIF1α Signaling*	0.0006	2.496	*HK3*,*MMP12*,*MMP25*,*MMP27*,*MMP9*,*NCF1*,*NCF2*,*PIK3CD*,*PIK3R1*,*PIK3R5*,*PLCG2*,*PRKCD*,*RAC3*,*SLC2A5*
*Natural Killer Cell Signaling*	0.0001	2.324	*CD244*,*CD48*,*IL18*,*JAK3*,*LCP2*,*MAP3K9*,*PIK3CD*,*PIK3R1*,*PIK3R5*,*PLCG2*,*PTPN6*,*RAC3*,*SYK*,*TYROBP*,*VAV1*
*Toll-like Receptor Signaling*	0.0001	2.236	*CD14*,*IL18*,*IL1A*,*LBP*,*TLR2*,*TLR4*,*TLR6*,*TLR8*,*TNF*
*Th2 Pathway*	2x10^-7^	2.138	*CCR1*,*CCR4*,*CCR5*,*CD28*,*CD40*,*CD86*,*CXCR6*,*ITGB2*,*JAK3*,*PIK3CD*,*PIK3R1*,*PIK3R5*,*SOCS3*,*SPI1*,*TNFRSF4*,*VAV1*
*VEGF Ligand-Receptor Interactions*	0.0002	2.121	*NRP2*,*PIK3CD*,*PIK3R1*,*PIK3R5*,*PLA2G2D*,*PLA2G2F*,*PLA2G5*,*PLCG2*,*PRKCD*
*IL-6 Signaling*	1.4x10^-5^	2.111	*A2M*,*CD14*,*CXCL8*,*IKBKE*,*IL18*,*IL1A*,*IL1R2*,*LBP*,*PIK3CD*,*PIK3R1*,*PIK3R5*,*SOCS3*,*TNF*
*PI3K/AKT Signaling*	0.0012	2.000	*CSF2RB*,*IFNAR1*,*IKBKE*,*IL10RA*,*IL1R2*,*IL21R*,*IL27RA*,*IL7R*,*ITGAM*,*ITGB2*,*JAK3*,*PIK3CD*,*PIK3R1*
*iNOS Signaling*	0.0054	2.000	*CD14*,*IKBKE*,*JAK3*,*LBP*,*TLR4*

Positive z scores indicate activation in C, negative ones indicate activation in HT. C: 30 mg of α-tocopheryl acetate/kg feed; HT: 30 mg α-tocopheryl acetate/kg and 1.5 mg hydroxytyrosol/kg.

#### Transcriptome comparison VE vs HT

The comparison of the transcriptome of animals receiving one of the two employed antioxidants yielded 90 DEGs, 28 being upregulated in HT and 62 being upregulated in VE (S4 Table). Highest expression changes were observed for *LIPN*, upregulated in HT (FC = 14.8x), and *RIMS3* (FC = 13.8x), upregulated in VE. Functional interpretation of the gene expression differences (S5 Table) was limited due to the moderate number of DEGs but suggested enrichment of *metabolic processes* (p_adj_ = 0.07).

#### Transcriptome comparisons involving VEHT group

When comparing C vs VEHT only *SLC30A2* and *SBK2* genes were detected as differentially expressed, both being upregulated in VEHT (S4 Table). Functional enrichment study could not be done due to the small number of DEGs.

Transcriptome comparison between VE and VEHT groups resulted in 192 DEGs (S4 Table), 52 being upregulated in VE and 140 being upregulated in VEHT. Functional enrichment results (S5 Table) were similar to those observed for the C vs VE contrast, with the genes upregulated in VE being mainly implicated in *lipid metabolism* and *lipid synthesis* (p_adj_ = 0.001 and 0.002, respectively), and those upregulated in VEHT showing a functional pattern resembling the control group, with enrichment of *immunity* (p_adj_ = 1x10^-14^) and *inflammatory response* (p_adj_ = 8x10^-10^) processes, *cytokine receptor interaction* (p_adj_ = 3.6x10^-10^), *chemokine activity* (p_adj_ = 8.4x10^-8^), *chemotaxis* (p_adj_ = 3.2x10^-7^) among many other related ontology terms and pathways. When we compare the results obtained in the contrast C vs VE (664 DEGs) and VE vs VEHT (192 DEGs), we found a majority of common DEGs (151 DEGs), most of them (132 DEGs) being upregulated in C in the first comparison and in VEHT in the second one.

The analysis of differential expression between the HT and the VEHT groups yielded 70 DEGs, with the majority of them (68 DEGs) being upregulated in VEHT (S4 Table) and involved in *inflammatory response* (p_adj_ = 1x10^-6^), *chemokine signalling* (p_adj_ = 7.7x10^-5^), *immune response* (p_adj_ = 5.4x10^-4^), *cytokine receptor interaction* (p_adj_ = 5.8x10^-4^) and other related functions and pathways (S5 Table). These results also resemble those observed in the C vs HT contrast, at a lower magnitude and significance level, but with VEHT group showing a functional profile similar to that of control animals, when compared to HT. When we compare the output of the C vs HT comparison (n = 587 DEGs) with that of HT vs VEHT comparison (70 DEGs) we found 66 common DEGs, most of them (65) being upregulated in C in the first comparison and in VEHT in the second one.

**Fig 4:**
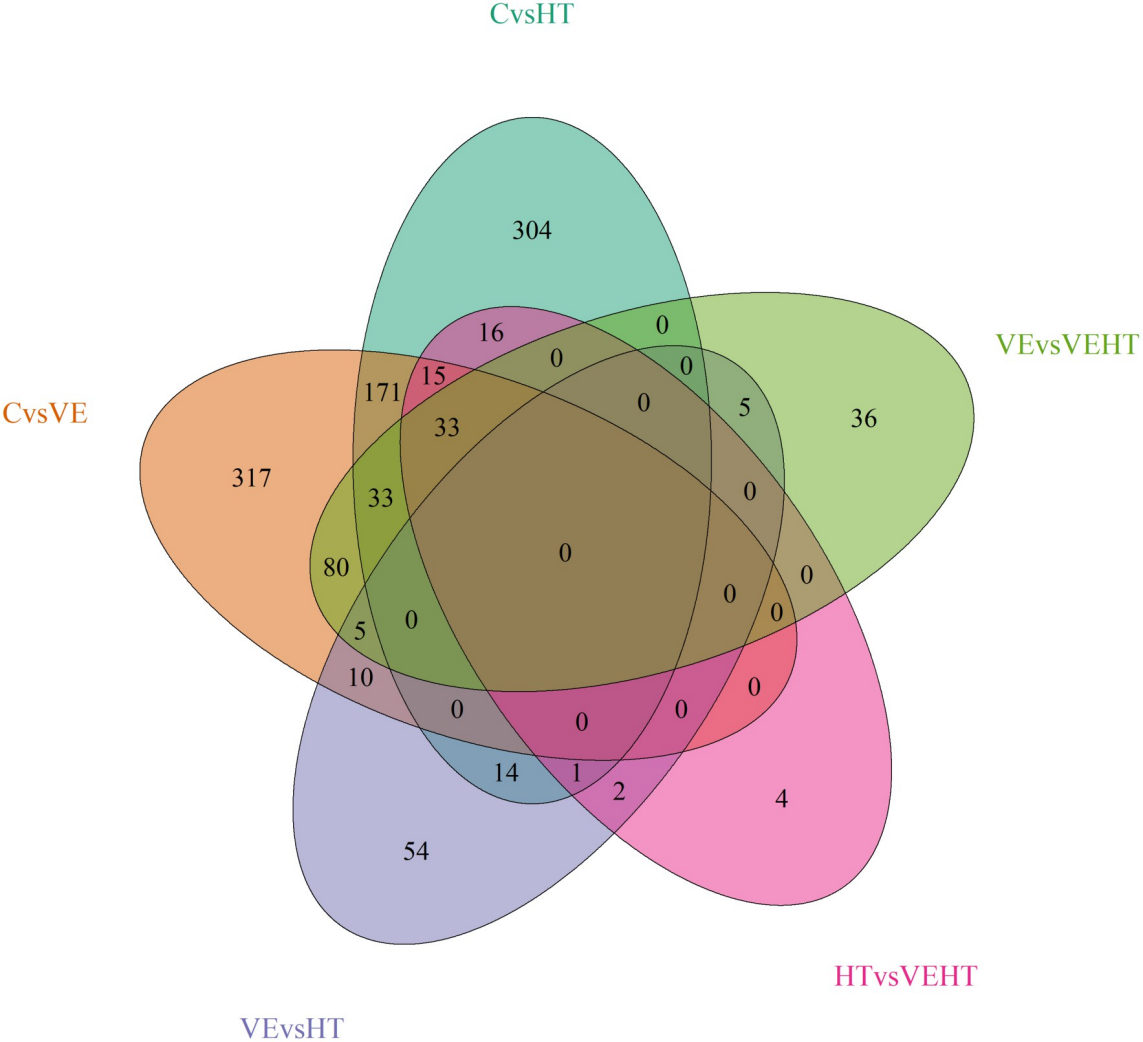
Venn diagram showing the overlapping of DEGs in the different transcriptome comparisons performed. The transcriptome comparison C vs VEHT has been excluded due to the very low number of DEGs.

When we compare the lists of DEGs detected after supplementing VE (with or without HT, C vs VE, n = 664 DEGs and HT vs VEHT, n = 70DEGs) we find 48 common DEGs, which are all upregulated in C in the first contrast and in VEHT in the second one. When we compare the effect of adding HT with low or high levels of VE (C vs HT, n = 587 DEGs and VE vs VEHT, n = 192DEGs) we find 66 common DEGs, all upregulated in C in the first contrast and in VEHT in the second one. These findings also support a similar behaviour of C and VEHT groups at the adipose transcriptome level in agreement with the abundant interaction effects found when a factorial analysis model was applied. Number of matching genes which are differentially expressed in the different transcriptome comparisons are shown in Fig 4.

### Prediction of regulators

Besides, IPA software was employed to predict potential upstream regulators involved in the expression changes observed in the two main transcriptome comparisons (C vs VE and C vs HT). Results are included in S7 Table, where regulators predicted to be activated in each treatment, with a z score higher than 2 (significantly activated in C) or lower than -2 (significantly activated in the antioxidant group, VE or HT), are listed. A large number of potential regulators were found, including transcription factors, cytokines, chemical drugs, transmembrane receptors, growth factors and other types of compounds and complexes.

**Fig 5:**
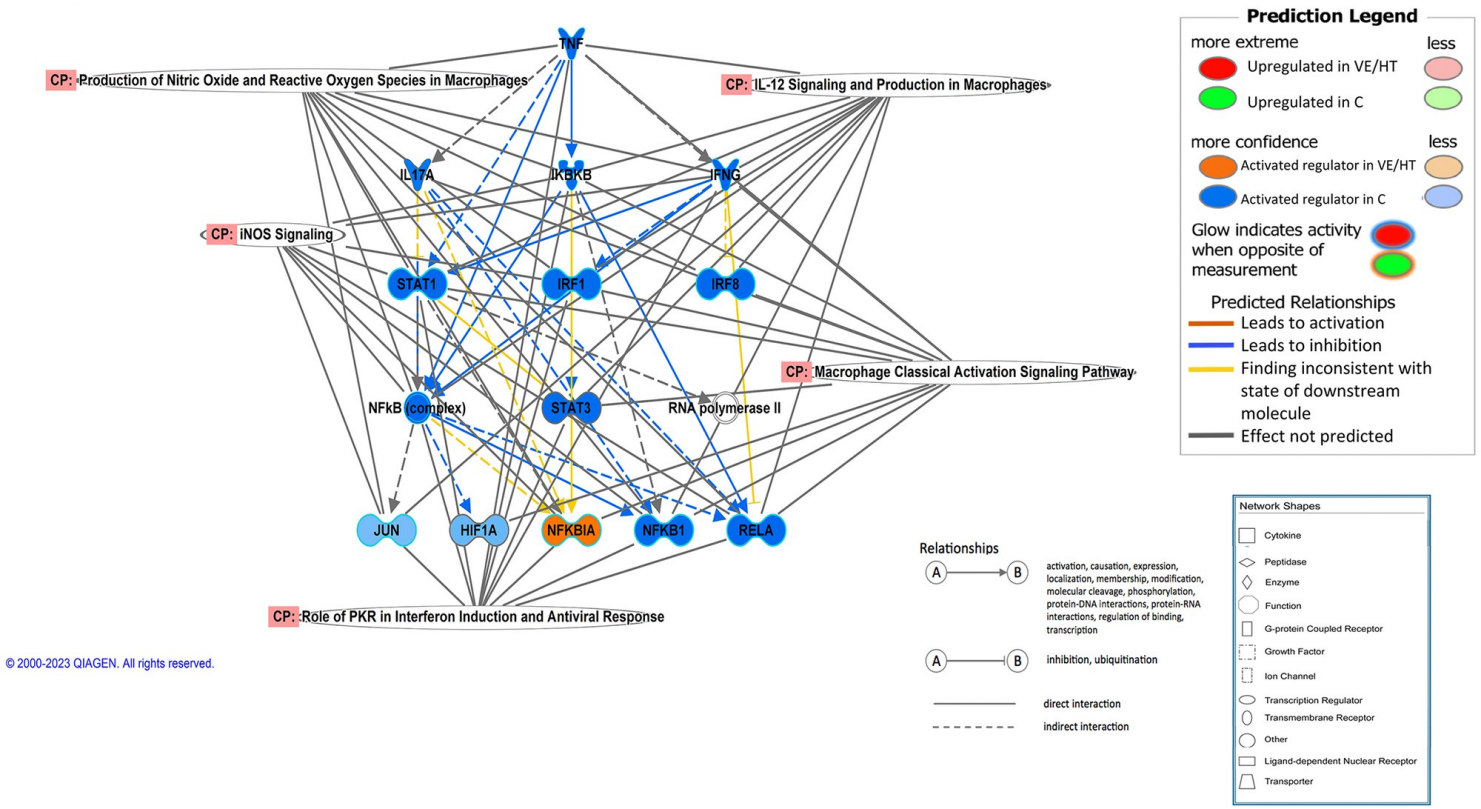
Regulatory causal network predicted by IPA as activated in C group in relation to VE or HT, and controlled by TNF. Blue nodes indicate regulators being predicted as activated in C.

The regulator molecule most significantly associated to the gene expression differences was *lipopolysaccharide* (p_adj_ = 4x10^-77^ and 4x10^-64^ in C vs VE and C vs HT, respectively), predicted as activated in C with the maximum z scores (10.13 and 9.35 in C vs VE and C vs HT, respectively). Also, there was a clear preponderance of proinflammatory cytokines being predicted to be activated in C group, in both comparisons. The cytokine with the highest activation score was Tumor necrosis factor (TNF), which was also differentially expressed, being upregulated in C (Fold Change = 4.3x and 5.2x in C vs VE and C vs HT, respectively). A regulatory causal network was predicted for this cytokine, which is shown in Fig 5, and is related to oxidative stress and macrophage activation pathways. This network related TNF with other relevant molecules and complexes essential in the development of inflammation, such as IFNG, NFKB, RELA, STAT1 or IRFs. These molecules were also predicted to be involved in the transcriptome effects observed for both contrasts.

Also, many proinflamatory interleukines (IL1, IL2, IL3, IL4, IL5, IL6, IL12, IL21, IL27 etc), interferons (IFNA1, IFNA2, IFNA4, IFNA5, IFNA6 among many others), interleukin and interferon receptors (IL1R, IL1R1, IL6R, IL17RA, IFNAR1) and interferon regulatory factors (IRF1, IRF3, IRF5, IRF7 and IRF8), were predicted as potential upstream regulators activated in C. Moreover, many of these molecules were upregulated in C, such as *IFNG*, *IRF8*, *IL1A*, *IL27* or *IL7R* among others.

**Fig 6:**
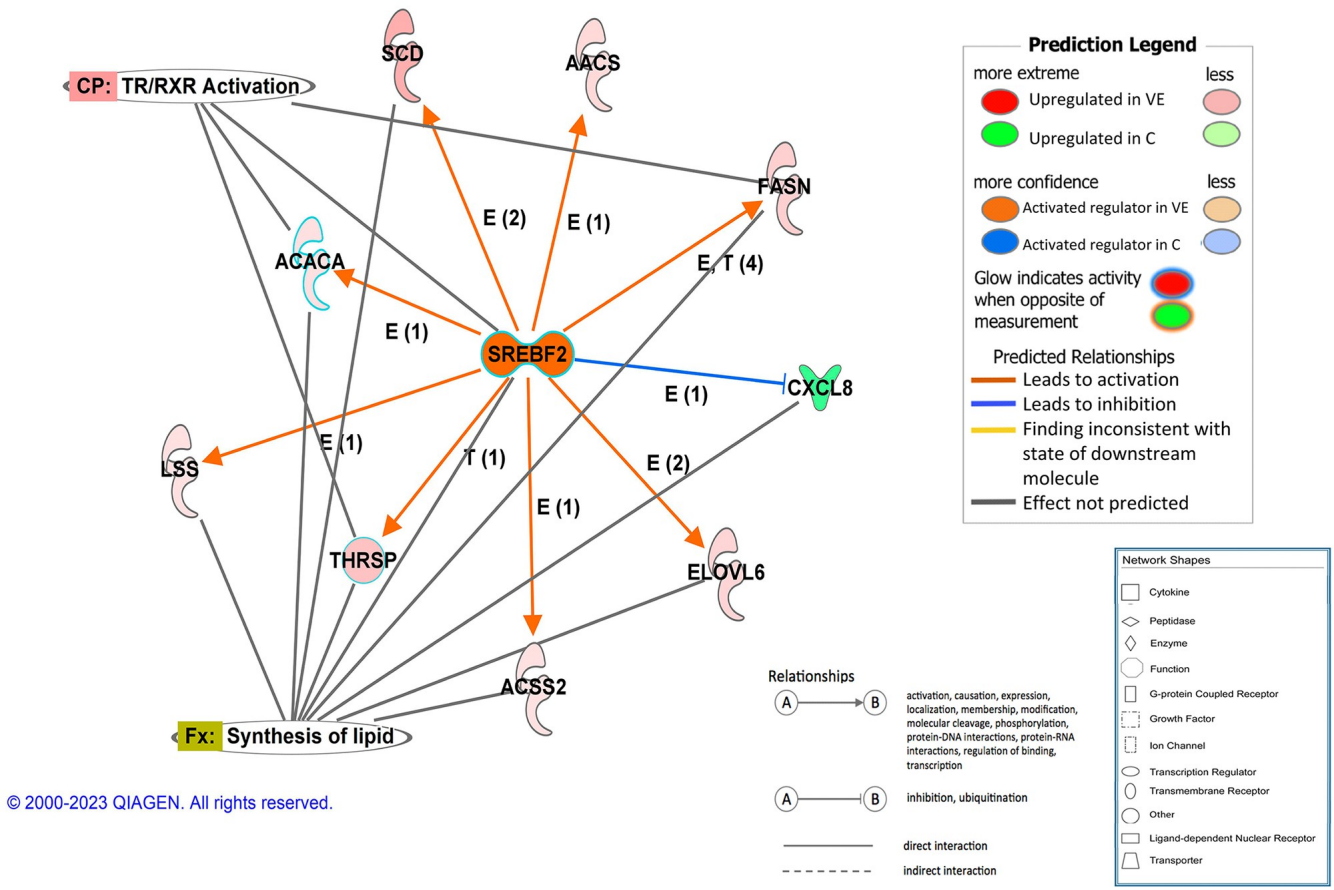
Regulatory causal network predicted by IPA as activated in VE group in relation to C, and controlled by SRBEF2. Orange nodes indicate molecules upregulated in VE. Green nodes indicate molecules upregulated in C.

Regarding the regulators predicted as being activated in the antioxidant-supplemented groups, there was a predominance of exogenous chemical drugs, mostly with immunosuppresor, immunomodulator, antiinflamatory or antioxidant effects. Within the large list of exogenous products, vitamin E is detected as potential regulator activated in both supplemented groups. Endogenous products identified as activated regulators in the antioxidant supplemented groups also included transcription factors. Some of them were predicted to be activated in both VE and HT groups in comparison to the control, and included CITED2, SIRT1, IKZF2, ZFP36, BACH2, CBX5, GFI1 and PRDM1. Also, there were transcriptional regulators detected only in one of the supplemented groups, such as FOXO4, ATF3, BTG2, CBL, RXRA, SREBF2, PPARG, PPARGC1A, or RARA among many others, detected in VE; and PAX1, NUPR1, KDM5B, MSD1 and others activated only in HT. A causal network was inferred for SREBF2, controlling lipid sinthesis and deposition, as shown in Fig 6. Cytokines were also detected, as interleukin 10 receptor subunit alpha (IL10RA, p_adj_ = 4x10^-23^ and 8x10^-18^ in VE and HT, respectively).

### Technical validation of differential gene expression

The relative expression of a selection of DEGs was quantified using Real-time RT-qPCR for the technical validation of RNA-Seq results. Genes selected for validation were *GPX1*, *SCD*, *JAZF1*, *NR4A3*, *LEP*, *CASP1*, *PON3* and *TLR2*. Pearson correlation was calculated between the expression values obtained from RNA-seq data (counts) and normalized gene expression data obtained by qPCR, with significant results for all the tested genes (correlation values between 0.828 and 0.999) (Table 3). Also, the concordance correlation coefficient (CCC) used to assess technical validation in high throughput transcriptomic studies [30] (CCC = 0.79), denoted an acceptable concordance between RNA-seq and qPCR expression values. Fold-Change values of differential expression and statistical significance tended to be greater when expression differences were analyzed by RNA-seq technology, in accordance with its higher sensitivity.

**Table 3:** Technical validation of RNA-seq results using qPCR: Genes, logarithm of fold change (log2 FC) (positive values correspond to upregulation in C group), statistical significance of the differential expression obtained with both techniques, Pearson correlation (r) between the two methodologies used and statistical significance of the correlation.

	**C vs HT**					
	**RNA-seq**		**qPCR**		**Correlation**	
Gene	log2 FC	*q value*	log2 FC	*p value*	*r*	*p value*
*TLR2*	3.337	0.010	2.432	0.140	0.996	3.5x10^-12^
*CASP1*	2.729	0.096	2.705	0.170	0.999	6.8x10^-17^
*GPX1*	1.574	0.040	1.831	0.059	0.967	2.6x10^-7^
*NR4A3*	0.323	0.769	0.301	0.431	0.875	1.9x10^-4^
*SCD*	-0.336	0.859	-0.106	0.879	0.950	2.2x10^-6^
*JAZF1*	-0.754	0.259	-0.632	0.027	0.884	1.3x10^-4^
*PON3*	-1.427	0.148	-1.361	0.081	0.949	2.3x10^-6^
*LEP*	-1.521	0.328	-1.686	0.188	0.985	5.5x10^-9^
	**C vs VE**					
	**RNA-seq**		**qPCR**		**Correlation**	
Gene	log2 FC	*q value*	log2 FC	*p value*	*r*	*p value*
*TLR2*	2.935	0.024	2.032	0.169	0.995	1.6x10^-11^
*CASP1*	3.243	0.023	2.682	0.172	0.999	4.6x10^-15^
*GPX1*	0.975	0.227	1.066	0.158	0.965	3.7x10^-7^
*NR4A3*	1.044	0.059	1.026	0.003	0.827	8.8x10^-4^
*SCD*	-3.186	2x10^-5^	-3.025	0.063	0.989	8.2x10^-10^
*JAZF1*	-1.099	0.007	-0.652	0.004	0.832	7.7x10^-4^
*PON3*	-1.966	0.001	-1.867	2x10^-4^	0.973	1.1x10^-7^
*LEP*	-1.874	0.035	-2.024	0.014	0.932	9.6x10^-6^

### Histological analyses

**Fig 7:**
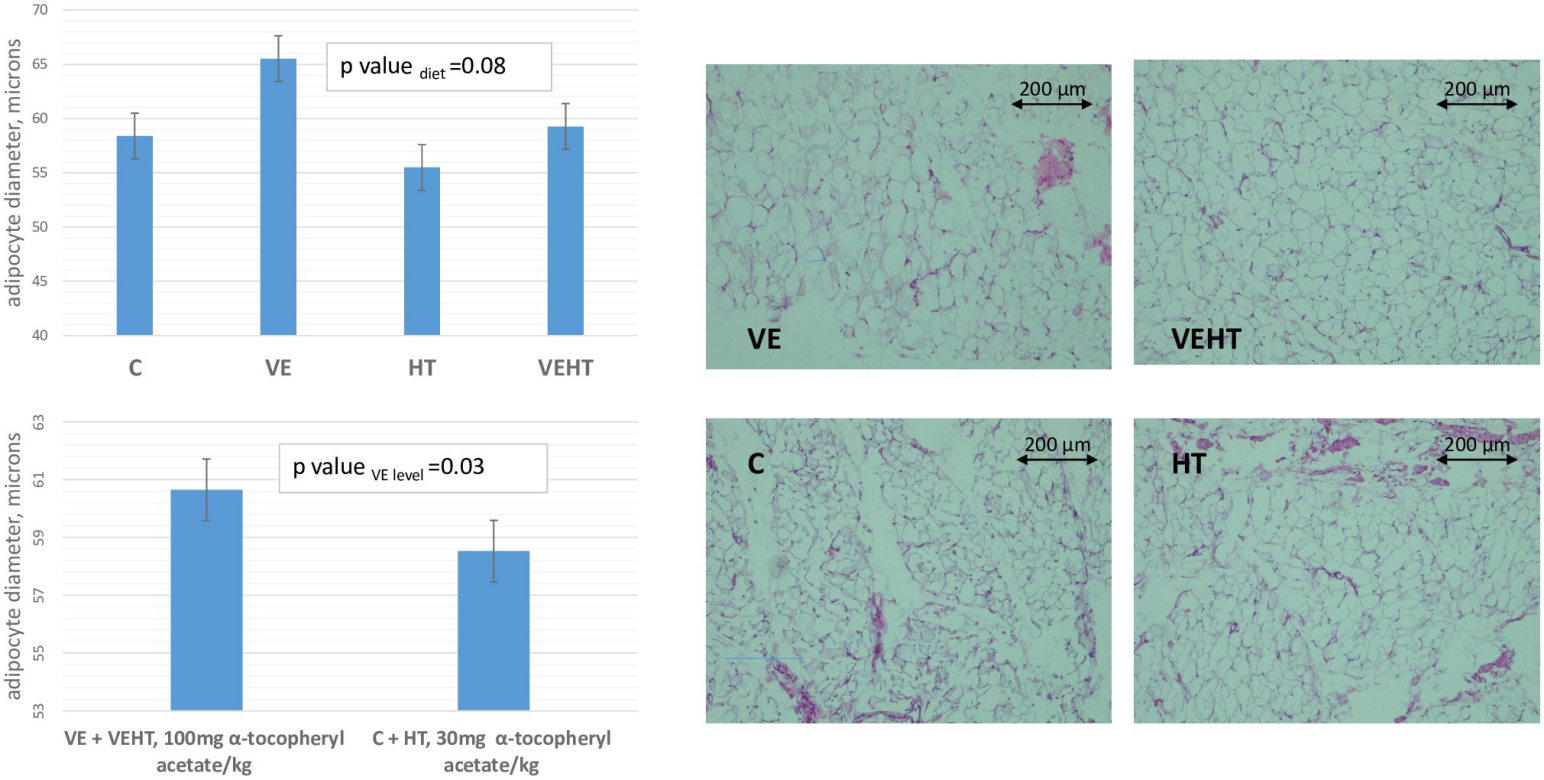
Adipocyte diameter observed in the adipose tissue of animals corresponding to different diet groups. C: 30 mg of α-tocopheryl acetate/kg feed (n = 12); VE: 100 mg of α-tocopheryl acetate/kg (n = 12); HT: 30 mg α-tocopheryl acetate/kg and 1.5 mg hydroxytyrosol/kg (n = 12); and VEHT: 100 mg/kg of α-tocopheryl acetate/kg and 1.5 mg hydroxytyrosol/kg feed (n = 12). VE+VEHT: animals receiving a high dose of vitamin E (100 mg of α-tocopheryl acetate/kg, VE and VEHT groups); C+HT: animals receiving a low dose of vitamin E (30 mg α-tocopheryl acetate/kg, C and HT groups). Photomicrographs of adipose tissue taken with a Leica ICC50W^™^ camera coupled to a Leica DM1000^™^ microscope corresponding to one sample from each experimental group.

The antioxidant supplementation had a suggestive effect on adipocyte diameter in adipose tissue (p-value = 0.08), with VE group showing the highest value and HT the lowest (Fig 7). When only the level of VE was considered, the size of the adipocytes was significantly larger in the animals born to sows receiving a high level of vitamin E (100 mg/kg, VE plus VEHT) vs the ones receiving 30 mg/kg (C plus HT) (60.6 microns versus 58.5 microns, respectively; p-value = 0.03).

## Discussion

In this work we have studied the effect of the inclusion level of antioxidant agents in Iberian sow’s perinatal diets on offspring adipose tissue function. For this purpose, adipose tissue transcriptome has been studied in the piglets at early postweaning, a critical development time when health is challenged and oxidative stress is exacerbated [31]. Phenotypic data obtained from the same animals employed in the present work [12–14] indicated a significant effect of the applied supplementations on oxidative stability in sows and in piglets’ plasma at day 20 of lactation and at the time of adipose tissue sampling, as well as on colostrum and milk composition.

A large number of DEGs were observed, especially after the individual supplementation of vitamin E or hydroxytyrosol (C vs VE and C vs HT transcriptome comparisons), with the results being validated by qPCR (Fig 1, Table 3). Functional interpretation of the transcriptome results suggests the development of a pro-inflammatory state in adipose tissue of Iberian piglets whose mothers received a basal level of vitamin E (30mg/kg) along the last third of gestation and lactation, in comparison to those receiving further individual antioxidant supplementation (Tables 1 and 2, Fig 5, S5 Table).

The observed upregulation of inflammatory and immune response genes, functions and pathways is not surprising, as Iberian pig is a local fat breed characterised by a profound adipogenic trend, altered lipid metabolism, obese phenotype and leptin resistance [7, 32], which are associated with the development of low-grade chronical inflammation in adipose tissue from early developmental stages [18]. This process known as “metaflammation” is observed in adipose tissue of obese individuals from different mammal species, where excessive lipid accumulation in adipocytes provokes immune cells infiltration, tissue remodelling, fibrosis, inflammation, oxidative stress, and ultimately insulin resistance [33, 34]. The process is characterised by the secretion of pro-inflammatory cytokines, especially interleukins (IL6, IL8, TNFα), chemokines (CCL2) and reduced secretion of adiponectin and IL10 [35]. Moreover, increased oxidative stress in fat tissue has been proven to be an early instigator and one of the important underlying causes of dysregulation of adipocytokines and development of metaflammation and metabolic syndrome [36, 37]. Thus the expected pro-oxidant environment in adipose tissue in C animals, in comparison to VE or HT-supplemented ones, may be contributing to the inflammatory profile.

The development of a proinflammatory status in the adipose tissue of C piglets, in comparison to those supplemented with vitamin E or hydroxytyrosol, is supported by the upregulation of key genes (*TNF*, *TGFB1*, *NFKB*, *NLRP3*, *ILs*, *CCLs*, *TLRs*), the enrichment of biological functions involved in immune and inflammatory responses; and the activation of many different interconnected signal transduction pathways (S4–S6 Tables).

Some of the identified pathways are associated to the cellular response to inflammation and its adaptation against deleterious consequences. For instance, *phagosome formation* was the canonical pathway with the highest activation z-score in control animals when compared to VE or HT (Tables 1 and 2). The role of phagosomes is eliminating harmful particles or cells to maintain tissue homeostasis [38], and are a key mechanism in innate immune response against pathogens. Nevertheless, in the scenario of adipose tissue biology, activation of phagosomes is an indication of the metabolic dysfunction, the macrophage infiltration, the effect of proinflammatory cytokines and the macrophage response for clearance of cell debris [39]. Phagosomes are thus part of an autophagy mechanism that digests apoptotic cell membranes and damaged cellular components in order to diminish inflammation. In fact, this pathway is narrowly related to another activated one, *inflammasome pathway*, as phagosomes can serve as a platform to control inflammasome activation, cytokine and chemokine expression and release. Another highly activated pathway in C animals was *Focal adhesion kinase* (FAK). FAK is a cytoplasmic protein-tyrosine kinase located at extracellular matrix which serves as a link between the extracellular environment and intracellular compartments and plays a central role in integrin signalling and adipocyte survival [40]. Indeed, adipocyte-specific FAK is essential for adipose tissue expansion and insulin sensitivity in obese genetic backgrounds, and it is known to be induced by oxidative stress [41]. Thus, the activation of these pathways in C animals could respond to an adaptive mechanism aimed at facilitating fat cell survival in obese individuals under stress conditions.

Other relevant activated pathways are directly involved in obesity pathogenesis and play key roles in adipose tissue inflammation and development of insulin resistance [42]. Some of these involve TREM1, MAPK, NFKB, PI3K, mTOR and different ILs signalling, which are largely interconnected (Tables 1 and 2). Triggering receptors expressed on myeloid cells 1 (TREM1) plays a significant role in the induction of inflammatory response in innate immunity, synergistically with other molecules such as Toll-like receptors (TLRs), and is involved in the pathogenesis of insulin resistance in obese individuals [43, 44]. In response to TREM receptor ligation, scaffolding proteins and downstream signalling molecules are activated, including phosphatidylinositol 3-kinase (PI3K). This activation triggers the kinase AKT, which, in turn activates mammalian target of rapamycin (mTOR), a key downstream target closely associated with the pathogenesis of obesity. In parallel, TLRs have a main role [45] and are known to regulate mitogen-activated protein kinase (MAPK) and nuclear factor-κB (NFKB) pathways, with *TLR2*, *TLR4*, *TLR6* and *TLR8* genes being upregulated in C (S4 Table). TLRs’ control MAPK regulation of TNFα-induced expression of inflammatory factors, with *TNF* being a key gene also upregulated in C group (Fig 5). TLRs stimulate NFKB and JNK signaling, which also lead to upregulation of the expression of inflammatory cytokines including TNFα and IL6, and further induce insulin resistance in adipocytes and macrophages [46, 47]. TLR activation also causes ROS production and mitochondrial failure [48], thus aggravating adipose tissue dysfunction.

Beside the cytokine storm unchained by adipose tissue macrophages, which are key players orchestrating low-grade chronic adipose tissue inflammation, there are other immune cell types within adipose tissue that play a role in starting, maintaining or trying to neutralize the inflammatory process, and which are also activated in C, specifically T cells and Natural Killer cells (Tables 1 and 2). *T helper type 1 (Th1) and 2 (Th2) activation pathways* showed the highest significance values for activation in C vs VE and HT, and several other canonical pathways involving T cells were identified too. Th1 and Th2 play an important role in mediating pro- and anti-inflammatory immune responses, respectively. Th1 cells produce Interferon γ (IFNG), a potent cytokine which is strongly upregulated in C vs VE (FC = 12x, S4 Table) and is essential in coordinating the inflammatory cascade and increasing macrophage accumulation [49]. IFNG also drives the transformation of macrophages from anti-inflammatory M2 to proinflammatory M1 cells. Natural killer (NK) cells are specialized innate lymphocytes that identify stressed fat cells and respond to this stress leading to proinflammatory activation of macrophages [50]. Also activated NK cells rapidly produce a variety of cytokines and cytotoxic products which induce apoptosis of target cells [51].

Obese individuals are characterised by increased levels of oxidative stress in adipose tissue [52]. This fact has been observed in different mammals including local fatty pig breeds [53] and specifically the Iberian pig [18]. In this experiment, adipose tissue transcriptome of piglets from the control group showed enrichment of functions and pathways related to the production of ROS and oxidative stress. In fact, two key pathways were activated in the C group when compared to either VE or HT groups (*Production of Nitric Oxide and Reactive Oxygen Species* and *iNOS Signaling*) (Tables 1 and 2, Fig 5). ROS are unstable reactive molecules which can oxidize important cellular constituents, thus having pro-oxidant and potentially harmful effects, but they can also play an important role in signal transduction cascades and normal cellular functions [54]. An adequate antioxidant system maintains the levels of ROS in a physiological range, but an imbalance between ROS production and antioxidant capacity causes ROS excess, which induces chemical modifications of DNA, protein, and lipids, leading to cell damage. Nitric oxide (NO) is a free radical known to limit the oxidative injury to mammalian cells mediated by ROS, by different mechanisms, and inducible nitric oxid synthase (iNOS) is one of the three isoforms of NOS which is only expressed when the cell is induced or stimulated, typically by pro-inflammatory cytokines and usually in oxidative environments [55]. These results suggest an imbalance between the production of ROS and their elimination by protective mechanisms, which is known to be related to chronic inflammation [33, 52]. Thus, the observed drastic activation of several processes and signalling pathways involved in inflammation suggests that the harmful oxidative activity of ROS, exacerbated in these genetically obese individuals, cannot be effectively counteracted by insufficient antioxidant compounds provided by the maternal control diet. This is in agreement with our previously published results, obtained from the same animals, showing a better oxidative status for the weaned piglets corresponding to supplemented groups in comparison to the control, reflected in higher plasma malondialdehyde levels in the latter [14].

Metabolic alterations occurring in adipose tissue of fatty animals (adipocyte dysfunction, inflammation and oxidative stress) are known to be associated with the release of a range of factors that predispose toward insulin resistance [56, 57]. Specifically, oxidative stress is known to alter insulin-mediated intracellular signalling pathways. In agreement, several pathways involving alterations in insulin signalling (*insulin secretion signalling*, *insulin receptor signalling*, *insulin resistance*, *Type I and Type II diabetes mellitus signalling pathways*) and others that have been found to enhance insulin resistance in obesity (such as the *adipocyte CREB/CRTC pathway*) [58] were activated in C (S6 Table). In fact, Iberian pigs are prone to obesity, leptin resistance, and insulin resistance [6, 7] and are considered a reliable translational model for studies on nutrition-associated metabolic diseases. Thus, although scientific evidences show inconsistent effects of antioxidant treatment on type II diabetes [59], our results support a potential relevant role of dietary antioxidants, applied at early developmental stages, for the prevention of insulin resistance and type II diabetes.

Supplemented animals showed enrichment of lipid synthesis, especially the animals receiving a high dose of vitamin E. In fact, some of the key genes involved in lipid synthesis and metabolism were upregulated in VE group in comparison to C, such as *SCD* (9.1x), *ELOVL6* (2.9x), *ME1* (2.5x), *LPL* (2.3x), *SREBF1* (1.9x) or *ACACA* (1.8x) (Figs 3 and 6). In HT-supplemented animals, this effect on lipid biosynthesis was not clearly observed, and, actually, some lipid-related genes such as *ME1*, *ELOVL6* or *FABP3* were upregulated in VE when compared to HT (S4 Table). In agreement, upstream regulators involved in adipogenesis and lipid synthesis were predicted to be activated in VE, but not in HT (S7 Table). The enrichment of lipid biosynthesis in supplemented animals may be associated with the decreased expression of lipogenic markers which is associated to inflammatory states [60], as chronic adipose tissue dysfunction compromises both adipogenesis and mature fat cell function. However, the strongest effect observed in VE group agrees with previous work indicating that vitamin E is able to regulate the expression of genes dependent on the nuclear receptor PPARγ [61] as well as to enhance adipocyte differentiation, lipid transport, adipogenesis, and mitochondrial function [62]. Moreover, results regarding expression of lipogenic genes were contrasted with histological data that confirmed adipocyte enlargement and higher lipid accumulation in adipocytes of vitamin E-supplemented piglets, which showed a higher diameter than control and hydroxytyrosol-supplemented animals (Fig 7). This finding is in agreement with a previous work showing that vitamin E supplementation reduces oxidative damage and adipose tissue fibrosis in obese mice; and allows adipocyte healthy expansion, increasing the storage capability and improving metabolic profile [62]. These matching results would indicate that VE-supplemented animals can achieve a healthy adipose tissue growth, because of an improved antioxidant status and extracellular matrix remodelling. Although there are histological studies comparing the adipocytes of fat and lean breeds [63], to the best of our knowledge this is the first study that compares the adipocytes’ size at the histological level between pigs whose mothers received different diets.

Other relevant pathways involved in tissue homeostasis were activated in animals supplemented with antioxidants. *Erythropoietin signalling pathway* was activated in both VE and HT groups (Tables 1 and 2). Erythropoietin is a glycoprotein hormone indispensable for erythropoiesis, with biological activities that extend to non-erythroid tissues, including antiapoptotic and anti-inflammatory effects in white adipose tissue [64]. *PTEN signalling pathway* was also activated in VE and to a lesser extent in HT. Phosphatase and tensin homologue (PTEN) modulates cell proliferation and is regulated by reversible oxidation of specific cysteines [65] and has an essential role in adipose tissue homeostasis, including both mass and distribution [66]. In fact, PTEN is inversely regulated and has contrary effects to MAPK and PI3K pro-inflammatory signal transduction pathways [42]. Moreover, the canonical pathway with the highest activation score in both VE and in HT groups, was *Antioxidant action of vitamin C*, implying an improved antioxidant stability in comparison to C group, again in concordance with previous findings on the same animals regarding better oxidative stability and homeostasis in supplemented groups [14].

As explained, results obtained in the two main comparisons (C vs VE and C vs HT) were similar according to the functional interpretation of the gene expression differences. In fact, there were 254 DEGs which were common to both transcriptome comparisons, and all of them showed the same regulation, with most of them being upregulated in C in the C vs VE comparison and also upregulated in C when comparing to HT (n = 244). In contrast, there were only ten common DEGs upregulated by the two antioxidants (*CYP2A19*, *COL4A62*, *RDH5*, *B3GNT4*, *RNF180*, *SLITRK6*, *ART4*, *MGP*, *PIK3R1*, *PCYT2*), suggesting a differentiated mechanism of action, which is expected due to their different nature and solubility.

Differential expression data allowed the inference of potential regulators (S7 Table) controlling the observed transcriptome profiles [35]. In agreement with the main findings, pro-inflammatory molecules are proposed as potential inductors of the genes upregulated in C, while products (mostly drugs) with anti-inflammatory or antioxidant effects are proposed to mediate or mimic the functional consequences observed in VE or HT. Prediction of lipopolysaccharide (LPS) as a potential regulator activated in C is coherent as LPS-induced signalling is known to contribute to the pro-inflammatory milieu in human obesity and to trigger the production of reactive oxygen and nitrogen species, such as through TLR4-mediated NADPH oxidase activation in macrophages [67]. A large number of known major mediators of adipose tissue inflammation and related to oxidative/antioxidant unbalance were predicted as activated in C, such as TNF, NFKB, TLR4, TLR2, PI3K, ERK, RELA, STAT1/3, IL1B or IL6 [68, 69], some of them being also differentially expressed (S4 Table). Also, several interferon and interferon receptors were identified in agreement with the regulatory role of interferon axis on inflammatory features in both myeloid cells and adipocytes, and its contribution to adipose tissue inflammation [70]. On the other hand, regulators related to oxidative stability and cell homeostasis were identified as activated in the antioxidant-supplemented groups. In fact, it is interesting to highlight that vitamin E was detected as potential regulator activated in the supplemented groups for both contrasts. One of the most significantly activated regulators in both antioxidant supplemented groups was interleukin 10 receptor subunit alpha (IL10RA). IL10 is an antiinflammatory cytokine that inhibits Th1 response by blocking the synthesis of proinflammatory cytokines at a transcriptional level, signaling through its receptors IL10RA and IL10RB [71]. Regarding transcription factors, SIRT1, known to be involved in the regulation of antioxidant genes and to protect the cells from ROS [72, 73], was predicted as activated in VE and in HT when compared to control group. Also, in VE, several transcription regulators involved in adipocyte differentiation and lipid metabolism were predicted as activated, such as RXRA, SREBF2, PPARG, PPARGC1A, or RARA, but these were not identified in the transcriptome comparison C vs HT. The results provide a large list of potential candidates to drive the antioxidant effects at the adipose tissue level, being potentially classified as AREs (antioxidant response elements) [74] but additional studies would be needed to identify the key molecules involved in the antioxidant protection effects of both types of supplemented products.

The transcriptome comparison between the combined treatment (VEHT) and the Control group yielded just two DEGs. This is an intriguing result, because the strong effect of both antioxidants when administered independently and the coincidence of genes modulated by them would suggest a potential additive effect when provided jointly, which is not at all observed. This finding is in agreement with the results obtained when a factorial model was applied, with scarce genes being affected by the global vitamin E or hydroxytyrosol levels, and a large number of genes showing a significant VE*HT interaction effect (S4 Table). Besides, the transcriptome comparisons between VE or HT groups against VEHT (S4 Table) support a pro-inflammatory transcriptome profile in VEHT, resembling that of the control group, and meaning that C and VEHT behave similarly at the adipose tissue transcriptome level in this experiment. In general, for performance traits, the combined treatment resulted in a similar effect to independent treatments [14]. Nevertheless, local effects of antioxidants at the adipose tissue level may be dependent on the specific role of ROS in this tissue. In fact, numerous previous studies indicate that ROS balance is required for normal functioning of white adipose tissue. Both, excessive but also diminished levels of ROS, which can result from over supplementation with antioxidants, can contribute to white adipose tissue dysfunction and inflammation, as well as insulin resistance [57]. Thus, it can be hypothesized that joint administration led to reduction of ROS below an adequate level to allow tissue homeostasis, and this is translated into a predicted altered state similar to that predicted for the control group. In addition, an antagonism between VE and HT could occur at the adipose tissue leading to the observed lack of effect on transcriptome. Further studies would be needed to clarify this unexpected but interesting result.

It has to be also considered that the nutrigenomic effects observed on piglet transcriptome in the present work are indirect, as they depend on the transmission of bioactive compounds from the supplemented mothers to the foetus via placenta, during gestation, and after birth through colostrum and milk. This implies that effects are modulated by the influence of the dietary antioxidants on the mother’s metabolism and on colostrum and milk composition. Prenatal transmission of bioactive compounds was not monitored, but colostrum and milk composition at days 7 and 20 lactation were studied in a previous work [12]. Vitamin content in colostrum and milk was modified by the applied treatments to a different extent. Briefly, supplementation with vitamin E increased the α-tocopherol content of colostrum and in a less extent that of milk, hydroxytyrosol did not influence α-tocopherol content but increased the content of vitamin A (retinol) in milk, and the combined supplementation showed the highest levels of vitamin E in colostrum and milk. This last finding may contribute to a very low level of ROS in VEHT group supporting the hypothesis presented in the previous paragraph. Also, antioxidant supplementation affected colostrum and milk fatty acid composition, with varying effects over time, but it is interesting to highlight that significant interaction effects were found, with milk obtained from C and VEHT sows at day 20 of lactation showing higher levels of n6-polyunsaturated fatty acids (PUFAs) than that of VE and HT sows. In the same way, oxidative stability of milk at day 20 of lactation was similar for C and VEHT groups. These findings are also relevant for the interpretation of the complex observed transcriptome results, as, for instance, n6-PUFA, which were increased in the milk of C and VEHT near weaning, are known to be prone to free radical-induced autoxidation and to contribute to tissue inflammation [75] and could help in the explanation of the similar transcriptome profile observed for C and VEHT.

## Conclusions

Maternal dietary supplementation with antioxidants during the perinatal period has a strong effect on the adipose tissue transcriptome of the offspring, studied in a critical development stage, five days after weaning. Results agree with an improved metabolic and antioxidant status of adipose tissue from piglets born to sows supplemented with either vitamin E or hydroxytyrosol. Animals from control group show gene expression changes that may be associated to an impaired adipose tissue homeostasis, with activation of oxidative stress pathways, immune signalling, and inflammation. Nutrigenomic effects induced by both antioxidants, when administered independently, are similar, but vitamin E activates lipid biosynthetic processes and increases adipocyte size and these effects are not observed after hydroxytyrosol supplementation.

The VEHT and C groups showed a similar transcriptome profile, with a negligible response observed after supplementing with both antioxidants jointly when compared to the control diet. This unexpected finding may indicate a local antagonism between either antioxidant agents or a detrimental effect of an excess of antioxidant supplementation on adipose tissue homeostasis. Effects of other bioactive compounds derived from the metabolic effects of the supplemented diets on the sows, and transferred to the piglets, cannot be discarded.

Potential regulators involved in the observed expression differences were detected, such as SIRT1, ATF3, TNF, IFNB, NFKB, STAT, RELA, IL1A, IL1B, CSF2 or TLR4, which could be related to the mechanisms of action of both antioxidants and the oxidative balance of adipose tissue.

## Supporting information

S1 Fig Principal component analyses of transcriptome data for the 24 analyzed samples. (TIF)

S1 Table Composition of experimental diets. (DOCX)

S2 Table Primers used for technical validation of differential expression. (XLSX)

S3 Table Significant interaction VE*HT effects (q<0.10). (XLSX)

S4 Table Lists of differentially expressed genes corresponding to each transcriptome contrast (q<0.10 and FC ≥1.5). (XLSX)

S5 Table Functional enrichment for the sets of DEGs obtained in each transcriptome contrast performed with DAVID tool. (XLSX)

S6 Table Prediction of canonical pathways activated or inhibited by the DEGs found in the comparisons CvsVE and CvsHT. (XLSX)

S7 Table Prediction of upstream regulators potentially involved in the expression changes found in the comparisons CvsVE and CvsHT. (XLSX)

## References

[1] Hanson M. The birth and future health of DOHaD. J Dev Orig Health Dis. 2015;6: 434–437. doi: 10.1017/S2040174415001129 26004094

[2] Li Q, Yang S, Chen F, Guan W, Zhang S. Nutritional strategies to alleviate oxidative stress in sows. Anim Nutr. 2022;9: 60–73. doi: 10.1016/j.aninu.2021.10.006 35949982 PMC9344312

[3] Hao Y, Xing M, Gu X. Research Progress on Oxidative Stress and Its Nutritional Regulation Strategies in Pigs. Anim Open Access J MDPI. 2021;11: 1384. doi: 10.3390/ani11051384 34068057 PMC8152462

[4] Vento M. Oxidative stress in the perinatal period. Free Radic Biol Med. 2019;142: 1–2. doi: 10.1016/j.freeradbiomed.2019.07.028 31351951

[5] Berchieri-Ronchi CB, Kim SW, Zhao Y, Correa CR, Yeum K-J, Ferreira ALA. Oxidative stress status of highly prolific sows during gestation and lactation. Animal. 2011;5: 1774–1779. doi: 10.1017/S1751731111000772 22440418

[6] Torres-Rovira L, Astiz S, Caro A, Lopez-Bote C, Ovilo C, Pallares P, et al. Diet-Induced Swine Model with Obesity/Leptin Resistance for the Study of Metabolic Syndrome and Type 2 Diabetes. Sci World J. 2012;2012: e510149. doi: 10.1100/2012/510149 22629144 PMC3354447

[7] Ovilo C, Fernández A, Fernández AI, Folch JM, Varona L, Benítez R, et al. Hypothalamic expression of porcine leptin receptor (LEPR), neuropeptide Y (NPY), and cocaine- and amphetamine-regulated transcript (CART) genes is influenced by LEPR genotype. Mamm Genome Off J Int Mamm Genome Soc. 2010;21: 583–591. doi: 10.1007/s00335-010-9307-1 21128076

[8] Gonzalez-Bulnes A, Torres-Rovira L, Ovilo C, Astiz S, Gomez-Izquierdo E, Gonzalez-Añover P, et al. Reproductive, endocrine and metabolic feto-maternal features and placental gene expression in a swine breed with obesity/leptin resistance. Gen Comp Endocrinol. 2012;176: 94–101. doi: 10.1016/j.ygcen.2011.12.038 22251656

[9] Gonzalez-Bulnes A, Ovilo C, Lopez-Bote CJ, Astiz S, Ayuso M, Perez-Solana ML, et al. Gender-specific early postnatal catch-up growth after intrauterine growth retardation by food restriction in swine with obesity/leptin resistance. Reproduction. 2012;144: 269–278. doi: 10.1530/REP-12-0105 22692087

[10] Blavi L, Solà-Oriol D, Llonch P, López-Vergé S, Martín-Orúe SM, Pérez JF. Management and Feeding Strategies in Early Life to Increase Piglet Performance and Welfare around Weaning: A Review. Animals. 2021;11: 302. doi: 10.3390/ani11020302 33503942 PMC7911825

[11] Vazquez-Gomez M, Garcia-Contreras C, Torres-Rovira L, Pesantez JL, Gonzalez-Añover P, Gomez-Fidalgo E, et al. Polyphenols and IUGR pregnancies: Maternal hydroxytyrosol supplementation improves prenatal and early-postnatal growth and metabolism of the offspring. PloS One. 2017;12: e0177593. doi: 10.1371/journal.pone.0177593 28545153 PMC5435224

[12] Laviano HD, Gómez G, Muñoz M, García-Casco JM, Nuñez Y, Escudero R, et al. Dietary Vitamin E and/or Hydroxytyrosol Supplementation to Sows during Late Pregnancy and Lactation Modifies the Lipid Composition of Colostrum and Milk. Antioxidants. 2023;12: 1039. doi: 10.3390/antiox12051039 37237905 PMC10215564

[13] Laviano HD, Gómez G, Escudero R, Nuñez Y, García-Casco JM, Muñoz M, et al. Maternal Supplementation of Vitamin E or Its Combination with Hydroxytyrosol Increases the Gut Health and Short Chain Fatty Acids of Piglets at Weaning. Antioxidants. 2023;12: 1761. doi: 10.3390/antiox12091761 37760063 PMC10526103

[14] Gómez G, Laviano HD, García-Casco JM, Escudero R, Muñoz M, Heras-Molina A, et al. Different Effect of Vitamin E or Hydroxytyrosol Supplementation to Sow’s Diet on Oxidative Status and Performances of Weaned Piglets. Antioxidants. 2023;12: 1504. doi: 10.3390/antiox12081504 37627499 PMC10451658

[15] O’Hea EK, Leveille GA. Significance of adipose tissue and liver as sites of fatty acid synthesis in the pig and the efficiency of utilization of various substrates for lipogenesis. J Nutr. 1969;99: 338–344. doi: 10.1093/jn/99.3.338 5350989

[16] Kershaw EE, Flier JS. Adipose tissue as an endocrine organ. J Clin Endocrinol Metab. 2004;89: 2548–2556. doi: 10.1210/jc.2004-0395 15181022

[17] Masschelin PM, Cox AR, Chernis N, Hartig SM. The Impact of Oxidative Stress on Adipose Tissue Energy Balance. Front Physiol. 2019;10: 1638. doi: 10.3389/fphys.2019.01638 32038305 PMC6987041

[18] Benítez R, Trakooljul N, Núñez Y, Isabel B, Murani E, De Mercado E, et al. Breed, Diet, and Interaction Effects on Adipose Tissue Transcriptome in Iberian and Duroc Pigs Fed Different Energy Sources. Genes. 2019;10: 589. doi: 10.3390/genes10080589 31382709 PMC6723240

[19] Benítez R, Nuñez Y, Ovilo C. Nutrigenomics in Farm Animals. J Investig Genomics. 2017;4: 00059. doi: 10.15406/jig.2017.04.00059

[20] Lange C. New NRC (2012) Nutrient Requirements of Swine. 2014. https://www.semanticscholar.org/paper/New-NRC-(-2012-)-Nutrient-Requirements-of-Swine-Lange/42fd9eb55172213163e8d4aa17852d1a302d2dde

[21] Rey AI, de-Cara A, Calvo L, Puig P, Hechavarría T. Changes in Plasma Fatty Acids, Free Amino Acids, Antioxidant Defense, and Physiological Stress by Oleuropein Supplementation in Pigs Prior to Slaughter. Antioxidants. 2020;9: 56. doi: 10.3390/antiox9010056 31936246 PMC7022758

[22] Garcia-Contreras C, Vazquez-Gomez M, Barbero A, Pesantez JL, Zinellu A, Berlinguer F, et al. Polyphenols and IUGR Pregnancies: Effects of Maternal Hydroxytyrosol Supplementation on Placental Gene Expression and Fetal Antioxidant Status, DNA-Methylation and Phenotype. Int J Mol Sci. 2019;20: 1187. doi: 10.3390/ijms20051187 30857182 PMC6429121

[23] FASTQC. A quality control tool for high throughput sequence data | BibSonomy. [cited 8 Nov 2023]. https://www.bibsonomy.org/bibtex/2b6052877491828ab53d3449be9b293b3/ozborn

[24] Kim D, Langmead B, Salzberg SL. HISAT: a fast spliced aligner with low memory requirements. Nat Methods. 2015;12: 357–360. doi: 10.1038/nmeth.3317 25751142 PMC4655817

[25] Anders S, Pyl PT, Huber W. HTSeq—a Python framework to work with high-throughput sequencing data. Bioinforma Oxf Engl. 2015;31: 166–169. doi: 10.1093/bioinformatics/btu638 25260700 PMC4287950

[26] Love MI, Huber W, Anders S. Moderated estimation of fold change and dispersion for RNA-seq data with DESeq2. Genome Biol. 2014;15: 550. doi: 10.1186/s13059-014-0550-8 25516281 PMC4302049

[27] Huang DW, Sherman BT, Lempicki RA. Systematic and integrative analysis of large gene lists using DAVID bioinformatics resources. Nat Protoc. 2009;4: 44–57. doi: 10.1038/nprot.2008.211 19131956

[28] Vandesompele J, De Preter K, Pattyn F, Poppe B, Van Roy N, De Paepe A, et al. Accurate normalization of real-time quantitative RT-PCR data by geometric averaging of multiple internal control genes. Genome Biol. 2002;3: RESEARCH0034. doi: 10.1186/gb-2002-3-7-research0034 12184808 PMC126239

[29] Steibel JP, Poletto R, Coussens PM, Rosa GJM. A powerful and flexible linear mixed model framework for the analysis of relative quantification RT-PCR data. Genomics. 2009;94: 146–152. doi: 10.1016/j.ygeno.2009.04.008 19422910

[30] Miron M, Woody OZ, Marcil A, Murie C, Sladek R, Nadon R. A methodology for global validation of microarray experiments. BMC Bioinformatics. 2006;7: 333. doi: 10.1186/1471-2105-7-333 16822306 PMC1539027

[31] Amazan D, Rey AI, Fernández E, López-Bote CJ. Natural vitamin E (d-α-tocopherol) supplementation in drinking water prevents oxidative stress in weaned piglets. Livest Sci. 2012;145: 55–62. doi: 10.1016/j.livsci.2011.12.022

[32] Lopez-Bote CJ. Sustained utilization of the Iberian pig breed. Meat Sci. 1998;49S1: S17-27. 22060709

[33] Martínez-Fernández L, Fernández-Galilea M, Felix-Soriano E, Escoté X, González-Muniesa P, Moreno-Aliaga MJ. Chapter 4—Inflammation and Oxidative Stress in Adipose Tissue: Nutritional Regulation. In: del Moral AM, Aguilera García CM, editors. Obesity. Academic Press; 2018. pp. 63–92.

[34] Ruiz-Ojeda FJ, Méndez-Gutiérrez A, Aguilera CM, Plaza-Díaz J. Extracellular Matrix Remodeling of Adipose Tissue in Obesity and Metabolic Diseases. Int J Mol Sci. 2019;20: 4888. doi: 10.3390/ijms20194888 31581657 PMC6801592

[35] Kawai T, Autieri MV, Scalia R. Adipose tissue inflammation and metabolic dysfunction in obesity. Am J Physiol—Cell Physiol. 2021;320: C375–C391. doi: 10.1152/ajpcell.00379.2020 33356944 PMC8294624

[36] Furukawa S, Fujita T, Shimabukuro M, Iwaki M, Yamada Y, Nakajima Y, et al. Increased oxidative stress in obesity and its impact on metabolic syndrome. J Clin Invest. 2004;114: 1752–1761. doi: 10.1172/JCI21625 15599400 PMC535065

[37] Manna P, Jain SK. Obesity, Oxidative Stress, Adipose Tissue Dysfunction, and the Associated Health Risks: Causes and Therapeutic Strategies. Metab Syndr Relat Disord. 2015;13: 423–444. doi: 10.1089/met.2015.0095 26569333 PMC4808277

[38] Fountain A, Inpanathan S, Alves P, Verdawala MB, Botelho RJ. Phagosome maturation in macrophages: Eat, digest, adapt, and repeat. Adv Biol Regul. 2021;82: 100832. doi: 10.1016/j.jbior.2021.100832 34717137

[39] Ju L, Han J, Zhang X, Deng Y, Yan H, Wang C, et al. Obesity-associated inflammation triggers an autophagy–lysosomal response in adipocytes and causes degradation of perilipin 1. Cell Death Dis. 2019;10: 1–16. doi: 10.1038/s41419-019-1393-8 30741926 PMC6370809

[40] Luk CT, Shi SY, Cai EP, Sivasubramaniyam T, Krishnamurthy M, Brunt JJ, et al. FAK signalling controls insulin sensitivity through regulation of adipocyte survival. Nat Commun. 2017;8: 14360. doi: 10.1038/ncomms14360 28165007 PMC5303880

[41] Lopez-Mejia IC, Pijuan J, Navaridas R, Santacana M, Gatius S, Velasco A, et al. Oxidative stress-induced FAK activation contributes to uterine serous carcinoma aggressiveness. Mol Oncol. 2023;17: 98–118. doi: 10.1002/1878-0261.13346 36409196 PMC9812840

[42] Wen X, Zhang B, Wu B, Xiao H, Li Z, Li R, et al. Signaling pathways in obesity: mechanisms and therapeutic interventions. Signal Transduct Target Ther. 2022;7: 298. doi: 10.1038/s41392-022-01149-x 36031641 PMC9420733

[43] Colonna M. The biology of TREM receptors. Nat Rev Immunol. 2023;23: 580–594. doi: 10.1038/s41577-023-00837-1 36750615 PMC9904274

[44] Subramanian S, Pallati PK, Sharma P, Agrawal DK, Nandipati KC. Significant association of TREM-1 with HMGB1, TLRs and RAGE in the pathogenesis of insulin resistance in obese diabetic populations. Am J Transl Res. 2017;9: 3224–3244. 28804542 PMC5553874

[45] Ospelt C, Gay S. TLRs and chronic inflammation. Int J Biochem Cell Biol. 2010;42: 495–505. doi: 10.1016/j.biocel.2009.10.010 19840864

[46] Peixoto LG, Teixeira RR, Vilela DD, Barbosa LN, Caixeta DC, Deconte SR, et al. Metformin attenuates the TLR4 inflammatory pathway in skeletal muscle of diabetic rats. Acta Diabetol. 2017;54: 943–951. doi: 10.1007/s00592-017-1027-5 28791487

[47] Korbecki J, Bajdak-Rusinek K. The effect of palmitic acid on inflammatory response in macrophages: an overview of molecular mechanisms. Inflamm Res Off J Eur Histamine Res Soc Al. 2019;68: 915–932. doi: 10.1007/s00011-019-01273-5 31363792 PMC6813288

[48] Okla M, Zaher W, Alfayez M, Chung S. Inhibitory Effects of Toll-Like Receptor 4, NLRP3 Inflammasome, and Interleukin-1β on White Adipocyte Browning. Inflammation. 2018;41: 626–642. doi: 10.1007/s10753-017-0718-y 29264745 PMC6066287

[49] Wang Q, Wang Y, Xu D. The roles of T cells in obese adipose tissue inflammation. Adipocyte. 2021;10: 435–445. doi: 10.1080/21623945.2021.1965314 34515616 PMC8463033

[50] Fernø J, Strand K, Mellgren G, Stiglund N, Björkström NK. Natural Killer Cells as Sensors of Adipose Tissue Stress. Trends Endocrinol Metab. 2020;31: 3–12. doi: 10.1016/j.tem.2019.08.011 31597606

[51] Bendelac A, Savage PB, Teyton L. The Biology of NKT Cells. Annu Rev Immunol. 2007;25: 297–336. doi: 10.1146/annurev.immunol.25.022106.141711 17150027

[52] Bondia-Pons I, Ryan L, Martinez JA. Oxidative stress and inflammation interactions in human obesity. J Physiol Biochem. 2012;68: 701–711. doi: 10.1007/s13105-012-0154-2 22351038

[53] Poklukar K, Čandek-Potokar M, Batorek Lukač N, Tomažin U, Škrlep M. Lipid Deposition and Metabolism in Local and Modern Pig Breeds: A Review. Animals. 2020;10: 424. doi: 10.3390/ani10030424 32138208 PMC7142902

[54] Sies H, Jones DP. Reactive oxygen species (ROS) as pleiotropic physiological signalling agents. Nat Rev Mol Cell Biol. 2020;21: 363–383. doi: 10.1038/s41580-020-0230-3 32231263

[55] Anavi S, Tirosh O. iNOS as a metabolic enzyme under stress conditions. Free Radic Biol Med. 2020;146: 16–35. doi: 10.1016/j.freeradbiomed.2019.10.411 31672462

[56] Lee B-C, Lee J. Cellular and molecular players in adipose tissue inflammation in the development of obesity-induced insulin resistance. Biochim Biophys Acta BBA—Mol Basis Dis. 2014;1842: 446–462. doi: 10.1016/j.bbadis.2013.05.017 23707515 PMC3800253

[57] Castro JP, Grune T, Speckmann B. The two faces of reactive oxygen species (ROS) in adipocyte function and dysfunction. Biol Chem. 2016;397: 709–724. doi: 10.1515/hsz-2015-0305 27031218

[58] Qi L, Saberi M, Zmuda E, Wang Y, Altarejos J, Zhang X, et al. Adipocyte CREB Promotes Insulin Resistance in Obesity. Cell Metab. 2009;9: 277–286. doi: 10.1016/j.cmet.2009.01.006 19254572 PMC2730923

[59] Park S, Park S-Y. Can antioxidants be effective therapeutics for type 2 diabetes? Yeungnam Univ J Med. 2020;38: 83–94. doi: 10.12701/yujm.2020.00563 33028055 PMC8016622

[60] Poulain-Godefroy O, Lecoeur C, Pattou F, Frühbeck G, Froguel P. Inflammation is associated with a decrease of lipogenic factors in omental fat in women. Am J Physiol Regul Integr Comp Physiol. 2008;295: R1–7. doi: 10.1152/ajpregu.00926.2007 18448614

[61] Landrier J-F, Marcotorchino J, Tourniaire F. Lipophilic Micronutrients and Adipose Tissue Biology. Nutrients. 2012;4: 1622–1649. doi: 10.3390/nu4111622 23201837 PMC3509510

[62] Alcalá M, Sánchez-Vera I, Sevillano J, Herrero L, Serra D, Ramos MP, et al. Vitamin E reduces adipose tissue fibrosis, inflammation, and oxidative stress and improves metabolic profile in obesity. Obesity. 2015;23: 1598–1606. doi: 10.1002/oby.21135 26148343

[63] Hausman GJ, Bergen WG, Etherton TD, Smith SB. The history of adipocyte and adipose tissue research in meat animals. J Anim Sci. 2018;96: 473–486. doi: 10.1093/jas/skx050 29385468 PMC6140974

[64] Alnaeeli M, Raaka BM, Gavrilova O, Teng R, Chanturiya T, Noguchi CT. Erythropoietin signaling: a novel regulator of white adipose tissue inflammation during diet-induced obesity. Diabetes. 2014;63: 2415–2431. doi: 10.2337/db13-0883 24647735 PMC4066343

[65] Cho S-H, Lee C-H, Ahn Y, Kim H, Kim H, Ahn C-Y, et al. Redox regulation of PTEN and protein tyrosine phosphatases in H2O2-mediated cell signaling. FEBS Lett. 2004;560: 7–13. doi: 10.1016/s0014-5793(04)00112-7 15017976

[66] Huang W, Queen NJ, McMurphy TB, Ali S, Cao L. Adipose PTEN regulates adult adipose tissue homeostasis and redistribution via a PTEN-leptin-sympathetic loop. Mol Metab. 2019;30: 48–60. doi: 10.1016/j.molmet.2019.09.008 31767180 PMC6812328

[67] Page MJ, Kell DB, Pretorius E. The Role of Lipopolysaccharide-Induced Cell Signalling in Chronic Inflammation. Chronic Stress. 2022;6: 24705470221076390. doi: 10.1177/24705470221076390 35155966 PMC8829728

[68] Eder K, Baffy N, Falus A, Fulop AK. The major inflammatory mediator interleukin-6 and obesity. Inflamm Res Off J Eur Histamine Res Soc Al. 2009;58: 727–736. doi: 10.1007/s00011-009-0060-4 19543691

[69] Han MS, White A, Perry RJ, Camporez J-P, Hidalgo J, Shulman GI, et al. Regulation of adipose tissue inflammation by interleukin 6. Proc Natl Acad Sci U S A. 2020;117: 2751–2760. doi: 10.1073/pnas.1920004117 31980524 PMC7022151

[70] Chan CC, Damen MSMA, Moreno-Fernandez ME, Stankiewicz TE, Cappelletti M, Alarcon PC, et al. Type I interferon sensing unlocks dormant adipocyte inflammatory potential. Nat Commun. 2020;11: 2745. doi: 10.1038/s41467-020-16571-4 32488081 PMC7265526

[71] Antuna-Puente B, Fellahi S, McAvoy C, Fève B, Bastard J-P. Interleukins in adipose tissue: Keeping the balance. Mol Cell Endocrinol. 2022;542: 111531. doi: 10.1016/j.mce.2021.111531 34910978

[72] Olmos Y, Sánchez-Gómez FJ, Wild B, García-Quintans N, Cabezudo S, Lamas S, et al. SirT1 regulation of antioxidant genes is dependent on the formation of a FoxO3a/PGC-1α complex. Antioxid Redox Signal. 2013;19: 1507–1521. doi: 10.1089/ars.2012.4713 23461683 PMC3797451

[73] Singh CK, Chhabra G, Ndiaye MA, Garcia-Peterson LM, Mack NJ, Ahmad N. The Role of Sirtuins in Antioxidant and Redox Signaling. Antioxid Redox Signal. 2018;28: 643–661. doi: 10.1089/ars.2017.7290 28891317 PMC5824489

[74] Raghunath A, Sundarraj K, Nagarajan R, Arfuso F, Bian J, Kumar AP, et al. Antioxidant response elements: Discovery, classes, regulation and potential applications. Redox Biol. 2018;17: 297–314. doi: 10.1016/j.redox.2018.05.002 29775961 PMC6007815

[75] Liput KP, Lepczyński A, Ogłuszka M, Nawrocka A, Poławska E, Grzesiak A, et al. Effects of Dietary n–3 and n–6 Polyunsaturated Fatty Acids in Inflammation and Cancerogenesis. Int J Mol Sci. 2021;22: 6965. doi: 10.3390/ijms22136965 34203461 PMC8268933

