# Supplementary figures and images for: Maternal dietary antioxidant supplementation regulates weaned piglets’ adipose tissue transcriptome and morphology
Source: PLoS One. 2024 Sep 12;19(9):e0310399. doi: 10.1371/journal.pone.0310399 (PMC11392410; doi:10.1371/journal.pone.0310399)

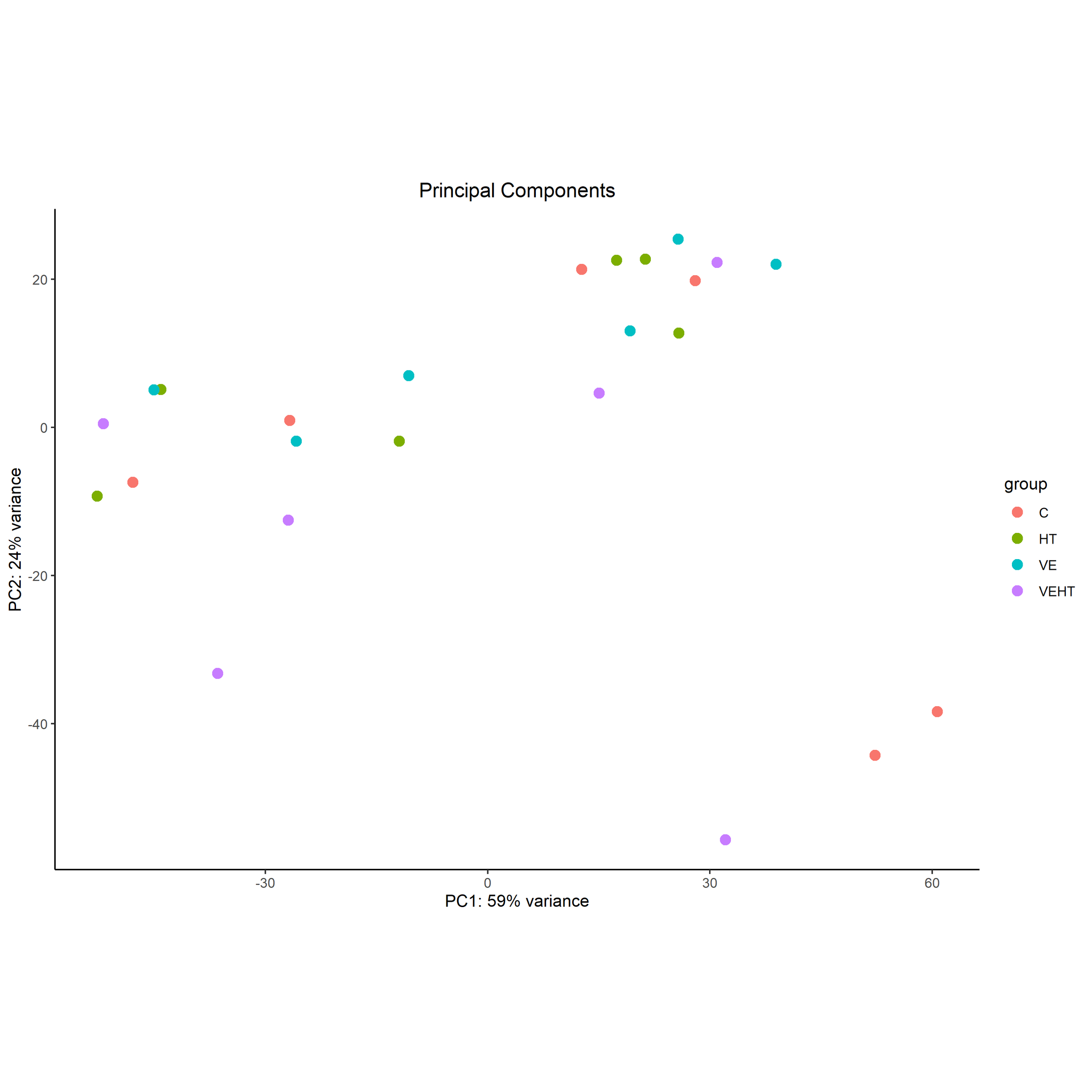

Supplement: S1 Fig — (TIF) [file pone.0310399.s001.tif]
