# Supplementary material for: Maternal dietary antioxidant supplementation regulates weaned piglets’ adipose tissue transcriptome and morphology
Source: PLoS One. 2024 Sep 12;19(9):e0310399. doi: 10.1371/journal.pone.0310399 (PMC11392410; doi:10.1371/journal.pone.0310399)
Supplement: S1 Table — (DOCX) [file pone.0310399.s002.docx]

**Table S1.**

1. Analyzed composition and fatty acid proportion of the sows’ experimental diets.

| ***Analyzed Composition* ^1^** | **CONTROL** | **VE** | **HT** | **HT + VE** |
| --- | --- | --- | --- | --- |
| Dry matter, % | 90.8 | 89.8 | 91.1 | 91.2 |
| Crude Protein, % | 13.1 | 14.0 | 15.0 | 14.5 |
| Fat, % | 4.3 | 3.6 | 3.8 | 3.7 |
| Ash, % | 6.5 | 6.4 | 6.9 | 6.0 |
| Fiber, % | 4.3 | 4.4 | 4.3 | 4.4 |
| Starch, % | 49.0 | 46.1 | 41.1 | 43.0 |
| Vitamin E, mg/kg | 70.5 | 103.6 | 65.4 | 114.1 |
| ***Fatty acid composition*** | |  |  |  |
| C14:0 | 0.60 | 0.66 | 0.56 | 0.60 |
| C16:0 | 19.80 | 21.48 | 19.10 | 20.11 |
| C16:1n-9 | 0.12 | 0.15 | 0.12 | 0.12 |
| C16:1n-7 | 0.79 | 0.87 | 0.73 | 0.78 |
| C18:0 | 4.77 | 4.80 | 4.45 | 4.54 |
| C18:1n-9 | 28.97 | 25.26 | 30.33 | 26.94 |
| C18:1n-7 | 1.92 | 1.49 | 1.57 | 1.60 |
| C18:2n-6 | 38.97 | 40.81 | 38.97 | 40.89 |
| C18:3n-3 | 3.04 | 3.44 | 3.15 | 3.36 |
| C20:0 | 0.30 | 0.26 | 0.30 | 0.31 |
| C20:1n-9 | 0.52 | 0.58 | 0.55 | 0.58 |
| ∑SAT | 25.47 | 27.20 | 24.41 | 25.55 |
| ∑MUFA | 32.33 | 28.35 | 33.29 | 30.02 |
| ∑PUFA | 42.01 | 44.25 | 42.11 | 44.25 |

^1^ Ingredients (%): Corn: 16.03; wheat: 10; Barley: 50; soya meal 47: 6.73; wheat bran: 6.54; pork lard: 1; beetroot pulp: 5; calcium carbonate: 1.98; dicalcium phosphate: 0.54; salt: 0.5; L-lysine: 0.50; Methionine: 0.04; threonine: 0.18; choline chloride: 0.03; premix: 0.3 (per kg/diet: vitamin A: 12,000 IU; Vitamin D_3_: 1400 IU; Vitamin B_1_: 1.1 mg; Vitamin B_2_: 6 mg; Vitamin B_12_: 0.08 mg; Vitamin B_6_: 12 mg; Nicotinic acid: 21 mg; Biotin: 0.12 mg; Pantothenic acid: 12 mg; Vitamin K_3_: 1.1 mg; Choline chloride: 225 mg; Fe (ferrous carbonate): 60 mg; Cu (pentahydrate sulphate): 14.2 mg; Zn (oxide): 100 mg; Mn (monohydrate sulphate): 30 mg; I (potassium iodure): 0.8 mg; Se (sodium selenite): 0.3 mg. Calculated metabolizable energy (ME) based on ingredient composition = 3030 Kcal ME/kg.

1. Analyzed composition of the piglets’ weaning diet

| ***Analyzed Composition*** | Weaning diet |
| --- | --- |
| Dry matter, % | 89.48 |
| Crude Protein, % | 14.46 |
| Fat, % | 4.68 |
| Fiber, % | 3.71 |
| Ash, % | 4.80 |
| Starch, % | 45.55 |
| Sugars, % | 4.09 |
| Met, % | 0.31 |
| Met+ Cys, % | 0.57 |
| Lys, % | 0.98 |
| Thr, % | 0.62 |
| Thp, % | 0.70 |
| Ca, % | 0.70 |
| P, % | 0.43 |
| ME (Kcal/kg) | 3250 |

Ingredients (%): Corn: 25; wheat: 20; barley: 30; fish protein: 5; soya 47: 8.71; beetroot pulp: 3; milk serum: 2.5; monocalcium phosphate: 0.42; calcium carbonate: 0.96; lard: 2.72; trn: 0.1; lys: 0.5; met: 0.06; salt: 0.3; enzymes: 0.1; protacid (60% formic acid+40% lignosulfonic acid): 0.3; choline chloride: 0.02; premix: 0.3.
